# Orexin receptor 2 agonist activates diaphragm and genioglossus muscle through stimulating inspiratory neurons in the pre-Bötzinger complex, and phrenic and hypoglossal motoneurons in rodents

**DOI:** 10.1371/journal.pone.0306099

**Published:** 2024-06-25

**Authors:** Ryuji Yamada, Tatsuki Koike, Masanori Nakakariya, Haruhide Kimura

**Affiliations:** 1 Neuroscience Drug Discovery Unit, Research, Takeda Pharmaceutical Company Limited, Fujisawa, Kanagawa, Japan; 2 Drug Metabolism and Pharmacokinetics Laboratory, Research, Takeda Pharmaceutical Company Limited, Fujisawa, Kanagawa, Japan; Showa University: Showa Daigaku, JAPAN

## Abstract

Orexin-mediated stimulation of orexin receptors 1/2 (OX[1/2]R) may stimulate the diaphragm and genioglossus muscle via activation of inspiratory neurons in the pre-Bötzinger complex, which are critical for the generation of inspiratory rhythm, and phrenic and hypoglossal motoneurons. Herein, we assessed the effects of OX2R-selective agonists TAK-925 (danavorexton) and OX-201 on respiratory function. In *in vitro* electrophysiologic analyses using rat medullary slices, danavorexton and OX-201 showed tendency and significant effect, respectively, in increasing the frequency of inspiratory synaptic currents of inspiratory neurons in the pre-Bötzinger complex. In rat medullary slices, both danavorexton and OX-201 significantly increased the frequency of inspiratory synaptic currents of hypoglossal motoneurons. Danavorexton and OX-201 also showed significant effect and tendency, respectively, in increasing the frequency of burst activity recorded from the cervical (C3–C5) ventral root, which contains axons of phrenic motoneurons, in *in vitro* electrophysiologic analyses from rat isolated brainstem–spinal cord preparations. Electromyogram recordings revealed that intravenous administration of OX-201 increased burst frequency of the diaphragm and burst amplitude of the genioglossus muscle in isoflurane- and urethane-anesthetized rats, respectively. In whole-body plethysmography analyses, oral administration of OX-201 increased respiratory activity in free-moving mice. Overall, these results suggest that OX2R-selective agonists enhance respiratory function via activation of the diaphragm and genioglossus muscle through stimulation of inspiratory neurons in the pre-Bötzinger complex, and phrenic and hypoglossal motoneurons. OX2R-selective agonists could be promising drugs for various conditions with respiratory dysfunction.

## Introduction

Respiration is a critical process involving the exchange of oxygen and carbon dioxide in the lung. The efficiency of this exchange is dependent on precise regulation of the diaphragm and genioglossus muscle, the main upper airway dilator muscle. Descending movement of the diaphragm into the abdominal cavity increases lung volume and induces inspiration [1], and anterior movement of the tongue by the genioglossus muscle widens the oropharyngeal airway [2]. Inspiratory neurons in the pre-Bötzinger complex, located in the ventrolateral medulla, regulate the coordinated activity of the diaphragm and genioglossus muscle by controlling the activity of phrenic motoneurons in the phrenic nucleus in the cervical spinal cord and hypoglossal motoneurons in the hypoglossal nucleus in the dorsomedial medulla, respectively [3], and are critical for the generation of inspiratory rhythm.

Dysregulation of the respiratory system is associated with various medical conditions, such as opioid-induced respiratory depression (OIRD) after surgery [4], opioid overdose [5], and sleep apnea [6], which is characterized by intermittent pauses in breathing during sleep. After surgery, opioids are applied to manage pain; however, their usage is associated with severe adverse events, including respiratory depression [7]. The opioid antagonist naloxone reduces these adverse events but also suppresses opioid analgesia. Moreover, because of life-threatening cardiovascular side effects, use of naloxone must be carefully considered [8]. Thus, drugs that suppress OIRD without compromising analgesia are required [9]. There are three types of sleep apnea: central sleep apnea (CSA), obstructive sleep apnea (OSA), and mixed apnea [10]. CSA is associated with blocked or weakened respiratory signals from the brainstem respiratory center to the diaphragm [6]. OSA is associated with obstruction of the upper airway [6]. Continuous positive airway pressure can maintain the airway in patients with sleep apnea by sending air through a mask on the nose and/or mouth and is widely used to treat both CSA and OSA. However, continuous positive airway pressure has limited adherence [11]. Although several compounds are reported to improve OSA [12, 13], currently no drugs are approved by the US Food and Drug Administration for the treatment of CSA and OSA. Therefore, novel drugs are needed.

Orexins (also called hypocretins) are neuropeptides produced in orexin neurons located in the lateral hypothalamus. The orexin peptides, orexin A (OX-A) and orexin B (OX-B), are produced by proteolytic cleavage of a single precursor peptide, prepro-orexin, in orexin neurons [14, 15]. Orexins regulate various physiologic functions through activation of two types of G protein–coupled receptors, orexin receptor 1 (OX1R) and orexin receptor 2 (OX2R); OX-A has similar agonistic activity for OX1R and OX2R, whereas OX-B has a moderate, approximately 40- to 50-fold, selectivity for OX2R over OX1R [14, 16]. Orexins are considered to be involved in the regulation of respiratory function via modulation of diaphragm and genioglossus muscle activity [17, 18]. In fact, orexin neurons project to the medulla of the brainstem, which contains the pre-Bötzinger complex and hypoglossal nucleus, and cervical spinal cord where the phrenic nucleus is located [19–21]. Moreover, OX-A, through activation of neurons in the pre-Bötzinger complex or phrenic motoneurons, increased electromyographic activity of the diaphragm in rats [19], and OX-B increased the frequency of burst activity recorded from the cervical ventral root, which contains phrenic motoneuron axons [22, 23], in rat isolated brainstem–spinal cord preparations [24]. OX-A also increased the firing rate of hypoglossal motoneurons in rat medullary slices and enhanced genioglossus muscle activity in rats [25]. In line with these observations, OX-A and OX-B increased minute ventilation in mice [26].

To understand the functional contribution of OX1R and OX2R in respiratory control, antagonists for OX1R (e.g., SB 334867) or OX2R (e.g., TCS OX2 29) have been evaluated in pre-clinical studies [25]. However, some of these antagonists have limited selectivity. For example, selectivity of SB334867 for OX1R over OX2R is only about 50-fold [27]. Therefore, detailed functional studies to assess each receptor’s contribution are warranted. For this objective, receptor-selective agonists that can penetrate the blood-brain barrier (BBB) are highly useful, because both OX-A and OX-B have limited OX1R/OX2R selectivity and poor BBB penetration [14, 16, 28].

In this study, we assessed the effects of OX2R-selective agonists TAK-925 (danavorexton) and OX-201 on respiratory function using OX-A as a reference. Danavorexton is a novel BBB-penetrable small-molecule OX2R agonist with >5000-fold selectivity over OX1R. Danavorexton produced potent arousal effects in rodents, non-human primates, healthy volunteers, and patients with narcolepsy type 1 or 2 [16, 29, 30]. OX-201 is a BBB-penetrable, OX2R-selective agonist tool suitable for *in vivo* studies using rodents due to its longer plasma half-life than danavorexton.

## Methods

### Animals

For *in vitro* electrophysiologic recording, neonatal Sprague-Dawley rats obtained from Charles River Laboratories Italia (Lecco, Italy) were used at postnatal day 1–10. For *in vivo* studies, we used male animals to prevent potential effects of female hormones on respiratory function [31]. Male C57BL/6J mice obtained from CLEA Japan (Tokyo, Japan) were used for electroencephalogram (EEG)/electromyogram (EMG) recordings at 13–14 weeks old (10 mice) and for pharmacokinetic studies at 8 weeks old (12 mice). OX2R knockout (KO) mice with a C57BL/6J genetic background were bred in our laboratory [16]. Male OX2R KO mice (8 mice) were used for EEG/EMG recordings at 20 weeks old. Male Sprague-Dawley rats obtained from Charles River Laboratories Japan (Kanagawa, Japan) were used for EEG/EMG recordings at 7–9 weeks old (8 rats), for EMG recordings of the diaphragm (32 rats) and genioglossus muscle (32 rats) at 7 weeks old, and for pharmacokinetic studies at 7–8 weeks old (25 rats). Male C57BL/6J mice obtained from Charles River Laboratories Italia (16 mice) were used for whole-body plethysmography recordings at 22–26 weeks old. All adult rats and mice were housed under laboratory conditions (12-h light/dark cycles, lights on at 07:00) with food (CE-2, CLEA Japan for animals obtained in Japan; rat and mouse maintenance diet Altromin R, A. Rieper, Bolzano, Italy, for animals obtained in Italy) and water available *ad libitum*. Animal health and behavior were monitored at least once a week.

*In vitro* electrophysiologic recordings in rats and whole-body plethysmography recordings in mice were conducted at Aptuit, LLC (Verona, Italy) in accordance with the Italian legislation and under authorization issued by the Italian Ministry of Health (protocol number: NT15: A50B0.N.ZHE and N 518/2019-PR). EEG/EMG recordings in C57BL/6J mice, OX2R KO mice, and rats, pharmacokinetic studies in mice and rats, and EMG recordings of the genioglossus muscle in rats were conducted at Takeda Pharmaceutical Company Limited under approval of the Institutional Animal Care and Use Committee of Shonan Health Innovation Park (protocol number: AU-00020488, AU-00030101, AU-00020629, AU-00020569, AU-00021176, AU-00020575, AU-00030384, and AU-00030552). EMG recordings of the diaphragm in rats were conducted at Axcelead Drug Discovery Partners, Inc. (Kanagawa, Japan), under approval of the Institutional Animal Care and Use Committee of Shonan Health Innovation Park (protocol number: AU-00030249). All surgery was performed under anesthesia, and all efforts were made to minimize suffering. The following criteria were used to determine when animals should be euthanized: more than 20% reduction of body weight, detachment of EEG/EMG electrodes, obvious abnormalities such as seizure. No animals met these criteria in this study. At the end of the experiments, all animals used in this study were euthanized; anesthetized rats used for EMG recordings of the diaphragm or genioglossus muscle were euthanized by exsanguination, and other animals were euthanized by carbon dioxide exposure. All animal experiments were performed in compliance with the Aptuit internal guidelines, guidelines approved by the Institutional Animal Care and Use Committee of Shonan Health Innovation Park, and ARRIVE guidelines.

### Chemicals and solutions

Danavorexton [16, 32] and OX-201, N-{(2S,3R)-4,4-difluoro-1-(2-hydroxy-2-methylpropanoyl)-2-[(2,3’,5’-trifluoro[1,1’-biphenyl]-3-yl)methyl] pyrrolidin-3-yl}methanesulfonamide, were synthesized by Takeda Pharmaceutical Company Limited. OX-A used for calcium mobilization assay was purchased from Peptide Institute, Inc. (Osaka, Japan). OX-A used for *in vitro* electrophysiology was purchased from Bachem (Bubendorf, Switzerland). For *in vitro* studies, all drugs were dissolved in dimethyl sulfoxide (DMSO) and diluted in each experimental solution. For EEG/EMG studies in C57BL/6J mice, OX2R KO mice, and rats, and for oral pharmacokinetic studies in C57BL/6J mice and rats, OX-201 was suspended in 0.5% (w/v) methylcellulose (MC) in distilled water (Fujifilm Wako Pure Chemical Corporation, Osaka, Japan). For whole-body plethysmography studies in mice, OX-201 was suspended in 0.5% (w/v) MC in distilled water (Merck Life Science, Milan, Italy), then orally administered to mice in a volume of 10 mL/kg of body weight, or to rats in a volume of 5 mL/kg of body weight. For EMG studies of the diaphragm and genioglossus muscle and pharmacokinetic studies in rats, OX-201 was solubilized in 0.1% (w/v) Polysorbate 80 and 20% (w/v) Captisol (CyDex Pharmaceuticals, KS, USA) solution and intravenously administered in a volume of 1 mL/kg of body weight.

### Calcium mobilization assay in human orexin receptor–expressing cells

Chinese hamster ovary-K1 (CHO-K1) cells (CCL-61; ATCC, VA, USA) stably expressing human OX1R or OX2R (hOX1R/CHO-K1 or hOX2R/CHO-K1 cells) were established as described previously [16]. Calcium mobilization in hOX1R/CHO-K1 or hOX2R/CHO-K1 cells was measured using an FDSS/μCELL system (Hamamatsu Photonics, Hamamatsu, Japan) as described previously [16]. The response to 0.5% DMSO and 100 nM OX-A (positive control) under 0.5% DMSO were used as 0% response and 100% response, respectively.

### *In vitro* assays for off-target profiling

The activity of OX-201 on 102 receptors, ion channels, and enzymes was evaluated at Eurofins Panlabs Discovery Services Taiwan (Taipei, Taiwan).

### Electrophysiologic recording of inspiratory neurons in the pre-Bötzinger complex and hypoglossal motoneurons in rat medullary slices

Neonatal rats (postnatal day 1–10) were deeply anesthetized by isoflurane, decapitated, and the neuraxis was isolated in a chamber filled with oxygenated artificial cerebrospinal fluid solution (ACSF; in mM: NaCl 125, KCl 2.5, MgCl_2_ 1, CaCl_2_ 2, NaHCO_3_ 25, NaH_2_PO_4_ 1.25, glucose 25; pH 7.4). The cerebellum was removed and the brainstem was mounted in a vibratome chamber (VT 1000S/1200S; Leica, Milan, Italy) filled with oxygenated gluconate cutting solution (in mM: K gluconate 130, KCl 15, EGTA 0.2, HEPES 20, glucose 25, kynurenic acid 2; pH 7.4). A 200–300 μm transverse section was cut using vibratome to remove the end rostral part, which revealed the caudal extent of the facial nucleus. Then serial 100–200 μm transverse sections were cut in the rostral to caudal direction. After observing the lateral loop of the principal inferior olive, transverse medullary slices containing the pre-Bötzinger complex and hypoglossal nucleus were cut (400- to 600-μm thick). The slices were then transferred into an oxygenated mannitol cutting solution (in mM: D-mannitol 225, glucose 25, KCl 2.5, NaH_2_PO_4_ 1.25, NaHCO_3_ 26, CaCl_2_ 0.8, MgCl_2_ 8, kynurenic acid 2; pH 7.4) at room temperature for 1 min as described previously [33]. This mannitol cutting solution has an intermediate ionic composition between gluconate cutting solution and ACSF and enable slices to be mildly recovered before their transfer into ACSF. Slices were transferred into ACSF solution at 30°C for 30 min and then to room temperature for 30 min. At least 30 min before the start of recording, slices were transferred into the recording solution in which the concentration of K^+^ was raised to 9 mM and concentration of Mg^2+^ was decreased to 0.5 mM to ensure the production of a long-term and stable rhythm. For the recording, the slice was transferred into a submerged recording chamber, where it was continuously perfused with fresh oxygenated modified ACSF solution (mACSF; in mM: NaCl 125, MgCl_2_ 0.5, KCl 9, CaCl_2_ 1, NaHCO_3_ 25, NaH_2_PO_4_ 1.25, glucose 25; pH 7.4) and maintained at 27 ± 2°C, with a flow rate of 2.3 ± 0.2 mL/min using a peristaltic pump.

The pre-Bötzinger complex is located ventral to the nucleus ambiguous [34]. In the pre-Bötzinger complex, multiple subpopulations of neurons create heterogenous neuronal networks [3, 35, 36]. In this study, neurons with rhythmic firing in the pre-Bötzinger complex were evaluated.

More than 90% of neurons in the hypoglossal nucleus are motoneurons [37]. Therefore, hypoglossal motoneurons were identified based on their location in the hypoglossal nucleus, characteristic size (20–60 μm in width), shape (multipolar neurons with a non-branching axon projecting ventrolaterally toward the hypoglossal nerve rootlets), and physiology (50–200 MΩ in input resistance) as previously reported [38, 39]. Following identification, neurons in the hypoglossal nucleus with rhythmic firing were evaluated as described previously [40].

In rat medullary slices, inspiratory neurons in the pre-Bötzinger complex are defined based on their rhythmic firing, which is in phase with inspiratory bursts of the hypoglossal nerve [41–43]. Because the hypoglossal nerve contains axons of hypoglossal motoneurons, we compared the frequency of rhythmic firing of pre-Bötzinger complex neurons with hypoglossal motoneurons. Under basal conditions, the mean ± standard error of the mean frequency of rhythmic firing of pre-Bötzinger complex neurons (0.14 ± 0.029 s^-1^, n = 8 neurons) was similar to that of hypoglossal motoneurons (0.12 ± 0.026 s^-1^, n = 5 neurons) (S1 Table). Moreover, these frequencies were similar to the frequency of inspiratory bursts of the hypoglossal nerve that is calculated from the results of previous reports (approximately 0.11–0.16 Hz) [42, 43]. Thus, in this study, we defined pre-Bötzinger complex neurons with rhythmic firing as inspiratory neurons. We also defined the rhythmic firing of inspiratory neurons in the pre-Bötzinger complex and hypoglossal motoneurons as inspiratory synaptic current.

Whole-cell voltage-clamp recordings were performed at a membrane potential of -60 and -55 mV for neurons in the pre-Bötzinger complex and hypoglossal motoneurons, respectively, using a borosilicate pipette filled with intracellular solution (in mM: K gluconate 114, KCl 6, MgATP 4, NaGTP 0.3, Na-Phosphocreatine 10, HEPES 10, EGTA 0.2; pH 7.25; osmolarity 300 mOsm with sucrose). Signals were acquired using Clampex 10.6 software (Axon; Molecular Devices, CA, USA) at a sampling rate of 10–20 kHz, and filtered at 2–10 kHz using an 8-pole Bessel filter built in the software and analyzed using Clampfit software (pClamp 10.6/10.7, Axon; Molecular Devices). Baseline and threshold levels for rhythmic firing, which was defined as inspiratory synaptic currents, were set using Clampfit software. Inspiratory synaptic currents were then visually detected, and the frequency, peak amplitude, duration, and area of inspiratory synaptic currents were automatically measured. The inspiratory synaptic currents of inspiratory neurons in the pre-Bötzinger complex and hypoglossal motoneurons detected in current signals consist of only negative currents from baseline (inward currents) [40, 42]. The area was calculated for the region of an inspiratory synaptic current that was below the baseline. Peak amplitude was measured as the maximum absolute value of an inspiratory synaptic current relative to the baseline. The duration was measured from the onset of an inspiratory synaptic current until its return to baseline. We excluded results in which the series resistance increased by >25% from the analysis of peak amplitude, duration, and area. We also excluded results in which neuronal firing was observed from the analysis of peak amplitude and area.

After 5 min of stabilization, neuronal activity under basal conditions was recorded for 2–12 min followed by recording in the presence of vehicle (0.1% DMSO) for 30 min. A single neuron was recorded in each slice. Mean values in the frequency of inspiratory synaptic currents during the last 1–2 min of basal conditions and those during the last 1–2 min of vehicle perfusion were compared to understand the effects of vehicle on neuronal activity. We confirmed that the perfusion of vehicle did not change the frequency of inspiratory synaptic currents of inspiratory neurons in the pre-Bötzinger complex and hypoglossal motoneurons (S1 Table). Thus, the effect of drugs on neuronal activity was assessed under vehicle (0.1% DMSO). To evaluate the effects of drugs on neuronal activity, after 5 min of stabilization, neuronal activity was recorded in the presence of vehicle for 2–20 min followed by recording after the application of drugs at 2–3 increasing concentrations (each for 4–15 min) per neuron. A single neuron was recorded in each slice. Mean values in the frequency, peak amplitude, duration, and area of inspiratory synaptic currents during the last 1–2 min in the presence of vehicle were used as control values, and those during the last 1–2 min with stimulation by each concentration of drug were used to calculate percent changes from control values.

### Electrophysiologic recording of burst activity from the cervical (C3–C5) ventral root, which contains phrenic motoneuron axons, in rat isolated brainstem–spinal cord preparations

Neonatal (postnatal day 1–4) rats were deeply anesthetized by isoflurane, decapitated, and the brainstem–spinal cord was dissected in a bath containing ACSF. The cerebellum and pons were ablated. At least 30 min before the start of recording, the tissue was transferred into the recording solution in which the concentration of K^+^ was raised to 5 mM and concentration of Mg^2+^ was decreased to 0.5 mM to ensure production of long-term and stable rhythm (mACSF, in mM: NaCl 125, MgCl_2_ 0.5, KCl 5, CaCl_2_ 1, NaHCO_3_ 25, NaH_2_PO_4_ 1.25, glucose 30; pH 7.4). The tissues were glued on coverslips, transferred into a recording chamber, and continuously perfused at 27 ± 2°C with mACSF at a flow rate of 2.3 ± 0.2 mL/min. Burst activity was measured in electrophysiological recordings from the cervical (C3–C5) ventral root, which contains phrenic motoneuron axons [22, 23], using a glass suction electrode. Signals were acquired in current-clamp configuration using Clampex 10.6 software (Axon; Molecular Devices) at a sampling rate of 10–20 kHz, and filtered at 2–10 kHz using an 8-pole Bessel filter built in the software. Acquired signals were filtered with a bandpass filter (0.01–3000 Hz), rectified, and integrated with a time-constant decay (50–200 ms) using LabChart software (AD Instruments, Dunedin, New Zealand). Rectification and integration were used for quantification of the burst activity because the spontaneous bursts detected in voltage signals consist of multiple positive and negative voltages from baseline [24]. Threshold levels for bursts were set using LabChart software (AD Instruments). Bursts were then automatically detected, and burst frequency, burst amplitude, burst duration, and burst area were measured. The burst area was calculated for the region of the burst that was above the baseline.

After 5 min of stabilization, burst activity from the cervical (C3–C5) ventral root under basal conditions was recorded for 2–20 min, followed by recording with vehicle (0.1% DMSO) for 30–40 min. Mean values in burst frequency during the last 2 min of basal conditions, and those during the last 2 min with vehicle perfusion, were used to understand the impact of vehicle on burst activity. We confirmed that the perfusion of vehicle did not change the burst frequency (S1 Table). Thus, the effect of drugs on neuronal activity was assessed under vehicle (0.1% DMSO). To evaluate the effects of drugs on burst activity, after 5 min of stabilization, burst activity from the cervical (C3–C5) ventral root in the presence of vehicle was recorded for 5–30 min, followed by recording with drug perfusion for 30–40 min until a plateau effect was achieved. The single concentration was tested in each tissue. Mean values in burst frequency, burst amplitude, burst duration, and burst area during the last 2 min in the presence of vehicle were used as control values, and those during the last 2 min of drug perfusion were used to calculate percent changes from control values.

### Pharmacokinetic study in C57BL/6J mice and rats

Blood samples from C57BL/6J mice were collected at various time points (0.25, 0.5, 1, 2, 4, 8, and 24 h) after oral administration of OX-201 (0.3, 1, 3, and 10 mg/kg). Blood samples from rats were collected at various time points after oral administration of OX-201 (3 mg/kg: 0.5, 1, 2, 3, 4, and 6 h; 10 and 30 mg/kg: 0.25, 0.5, 1, 2, 4, 8, and 24 h) or intravenous administration of OX-201 (0.3, 1, and 3 mg/kg: 0.083, 0.17, 0.25, 0.5, and 1 h). Plasma was separated from the blood samples by centrifugation. The concentrations of drugs in the plasma were quantified with high-performance liquid chromatography-tandem mass spectrometry. The lower limit of quantitation was 3 ng/mL. The maximum concentration (C_max_), time to reach C_max_ (T_max_), and mean residence time (MRT) were calculated.

### EEG/EMG recording in C57BL/6J mice and OX2R KO mice

Implantation of EEG/EMG electrodes and EEG/EMG recordings were performed as described previously [16]. Vehicle (0.5% [w/v] MC in distilled water) or OX-201 was orally administered at zeitgeber time (ZT) 5 (lights on at ZT0), the resting period in nocturnal rodents when orexin neurons are least active [44]. EEG/EMG signals were amplified, filtered (EEG, 0.5–250 Hz; EMG, 16–250 Hz), and digitized at a sampling rate of 200 Hz using VitalRecorder software (Kissei Comtec, Nagano, Japan). Locomotor activity was measured by an infrared activity sensor (Biotex, Kyoto, Japan). SleepSign software (Kissei Comtec) was used to automatically classify wakefulness, non-rapid eye movement (NREM) sleep, or rapid eye movement (REM) sleep in 4-s epochs, based on EEG/EMG/locomotor activity data. Each stage was characterized as: (1) wakefulness, low-amplitude EEG with high-voltage EMG activity or high locomotion score; (2) NREM sleep, high-amplitude slow-wave EEG with low-voltage EMG activity; and (3) REM sleep, theta-dominated EEG with EMG atonia. The study was conducted with a crossover design and time spent in wakefulness was calculated. The duration of the experiment was approximately 1 month for C57BL/6J mice and approximately 3 weeks for OX2R KO mice.

### EEG/EMG recording in rats

Rats were anesthetized by an intraperitoneal injection of a mixture of medetomidine (0.15 mg/kg; Nippon Zenyaku Kogyo, Tokyo, Japan), midazolam (2 mg/kg; Astellas Pharma, Tokyo, Japan), and butorphanol (2.5 mg/kg; Meiji Seika Pharma, Tokyo, Japan) and fixed to stereotaxic apparatuses (Kopf, CA, USA). A telemetric transmitter (TL11M2-F20-EET, Data Sciences International, Inc., St. Paul, MN, USA) was implanted under sterile conditions to record EEG, EMG of the dorsal neck muscle, and locomotor activity. EEG electrode leads were placed over the left frontal cortex (2.2 mm anterior and 3 mm lateral to the bregma) and left parietal cortex (1 mm anterior and 3 mm lateral to the lambda), and electrodes fixed to the skull with dental cement. After at least a 1-week recovery period in home cages, the rats were habituated to the recording chambers. Vehicle (0.5% [w/v] MC in distilled water) or OX-201 was orally administered at ZT5, the resting period in nocturnal rodents when orexin neurons are least active [44]. EEG, EMG, and locomotor activity were recorded using the telemetry system (Dataquest ART system, Data Sciences International, Inc.). EEG/EMG signals were amplified, filtered (EEG, 0.1–250 Hz; EMG, 0.1–250 Hz), and digitized at a sampling rate of 250 Hz using Dataquest ART software (Data Sciences International, Inc.). SleepSign software (Kissei Comtec) was used to automatically classify wakefulness, NREM sleep, or REM sleep in 4-s epochs, based on EEG/EMG/locomotor activity data. Each stage was characterized as: (1) wakefulness, low-amplitude EEG with high-voltage EMG activity or high locomotion score; (2) NREM sleep, high-amplitude slow-wave EEG with low-voltage EMG activity; and (3) REM sleep, theta-dominated EEG with EMG atonia. The study was conducted with a crossover design and time spent in wakefulness was calculated. The duration of the experiment was approximately 1 month.

### EMG recording of the diaphragm in anesthetized rats

Rats were anesthetized with isoflurane (induction, 2%; maintenance, 1.5%) with anesthetic depth assessed by reflex responses to paw pinches. The tail vein was cannulated for intravenous drug administration. Rats were laid on disposable body warmers to prevent decreases in body temperature. The diaphragm was exposed, then two needle electrodes were implanted into the costal diaphragm to monitor diaphragm EMG (differential recording). After 10 min of baseline recording, vehicle (0.1% [w/v] Polysorbate 80 and 20% [w/v] Captisol solution) or OX-201 was intravenously administered, and then EMG was recorded for 10 min. EMG was amplified using Bio Amp system (ML132; AD Instruments), high-pass filtered (10 Hz), digitized using PowerLab software (AD Instruments), and then rectified and integrated with a time-constant decay (100 ms) using LabChart software (AD Instruments). Threshold levels for bursts were set using LabChart software (AD Instruments). Bursts were then automatically detected, and burst frequency, burst amplitude, and tonic activity were measured. Tonic activity was defined as the mean value of integrated EMG signals between bursts. Burst amplitude and tonic activity were averaged in 1-min bins for the following analysis. Mean values in burst frequency, burst amplitude, and tonic activity during 2 min before vehicle or OX-201 administration were used as control values, and those during 10 min after vehicle or OX-201 administration were used to calculate percentages of control values.

### EMG recording of the genioglossus muscle in anesthetized rats

EMG recording of the genioglossus muscle was conducted as described previously [25] with slight modifications. Rats were anesthetized with urethane (2.0 g/kg, intraperitoneal injection) with anesthetic depth assessed by reflex responses to paw pinches. The tail vein was cannulated for intravenous drug administration. Each rat was laid on a servo-controlled electric heating pad. Rat body temperature was monitored with the heating pad’s rectal probe and kept at approximately 37°C. Vagus nerves were sectioned bilaterally to increase genioglossus muscle activity [25, 45]. Rats were tracheotomized and the genioglossus muscle exposed. Then two needle electrodes were implanted bilaterally into the genioglossus muscle to monitor EMG (differential recording). After 10 min of baseline recording, vehicle (0.1% [w/v] Polysorbate 80 and 20% [w/v] Captisol solution) or OX-201 was intravenously administered and then EMG was recorded for 10 min. EMG was amplified 1000-fold, bandpass filtered (100–5000 Hz) using Microelectrode AC Amplifier (Model 1800; A-M systems, Carlsborg, WA, USA), digitized using AD converter (Power 1401; Cambridge Electronic Design, Cambridge, UK), acquired using Spike2 software (Cambridge Electronic Design), and then rectified and integrated with a time-constant decay (100 ms) using LabChart software (AD Instruments). Threshold levels for bursts were set using LabChart software (AD Instruments). Bursts were then automatically detected, and burst frequency, burst amplitude, and tonic activity were measured. Tonic activity was defined as the mean value of integrated EMG signals between bursts. Burst amplitude and tonic activity were averaged in 1-min bins for the following analysis. Mean values in burst frequency, burst amplitude, and tonic activity during 2 min before drug administration were used as control values, and those during 10 min after vehicle or OX-201 administration were used to calculate percentages of control values.

### Whole-body plethysmography recording in mice

Respiratory activity was measured in free-moving mice using whole-body plethysmography (Buxco Small Animal Whole Body Plethysmography; Data Sciences International, Inc., New Brighton, MN, USA). First, mice were placed in whole-body plethysmography chambers at ZT2 for 1 h for 3 consecutive days to habituate to the chambers. The effects of OX-201 on respiratory activity were investigated by crossover design with an interval of ≥3 days between treatments. Each session consisted of 2 days. On the first day, mice were placed in the chambers at ZT2, handled to simulate administration at ZT5, and kept in the chamber by ZT9. On the second day, mice were again placed in the chambers at ZT2, administered vehicle or OX-201 at ZT5, which is the resting period in nocturnal rodents when orexin neurons are least active [44], then respiratory signals were recorded. Recorded signals were analyzed for respiratory rate (number of breaths for 1 min), tidal volume (the amount of air that moves in and out of lungs with each respiratory cycle), minute volume (total sum of volume delivered over 1 min, attained by multiplying respiratory rate by tidal volume), peak inspiratory flow (maximum inspiratory flow in one breath), peak expiratory flow (maximum expiratory flow in one breath), inspiratory time (time spent inhaling during each breath), and expiratory time (time spent exhaling during each breath). Respiratory parameters were averaged in 1-h bins for the following analysis and the mean values during 3 h after vehicle or OX-201 administration were calculated. The duration of the experiment was approximately 1 month.

### Statistical analysis

Results are presented as mean ± standard error of the mean. For *in vitro* electrophysiologic analyses, comparison between groups were performed on raw data. The statistical significance between two groups was determined by two-tailed paired *t*-test with closed testing procedure from the highest drug concentration. In the EEG/EMG study of OX2R KO mice, two-tailed paired *t*-test was used to assess the significance of pairwise differences between groups. For other studies, Dunnett’s multiple comparison test was used to assess the significance of the differences compared with vehicle groups. P values < 0.05 were considered statistically significant. Statistical analyses were conducted using EXSUS software (version 8.0.0; CAC EXICARE, Tokyo, Japan). Graphs were obtained with GraphPad (version 9) software. Values for 50% effective concentrations (EC_50_) were obtained with GraphPad (version 9) software.

## Results

### Characterization of a novel OX2R-selective agonist, OX-201

**Fig 1:**
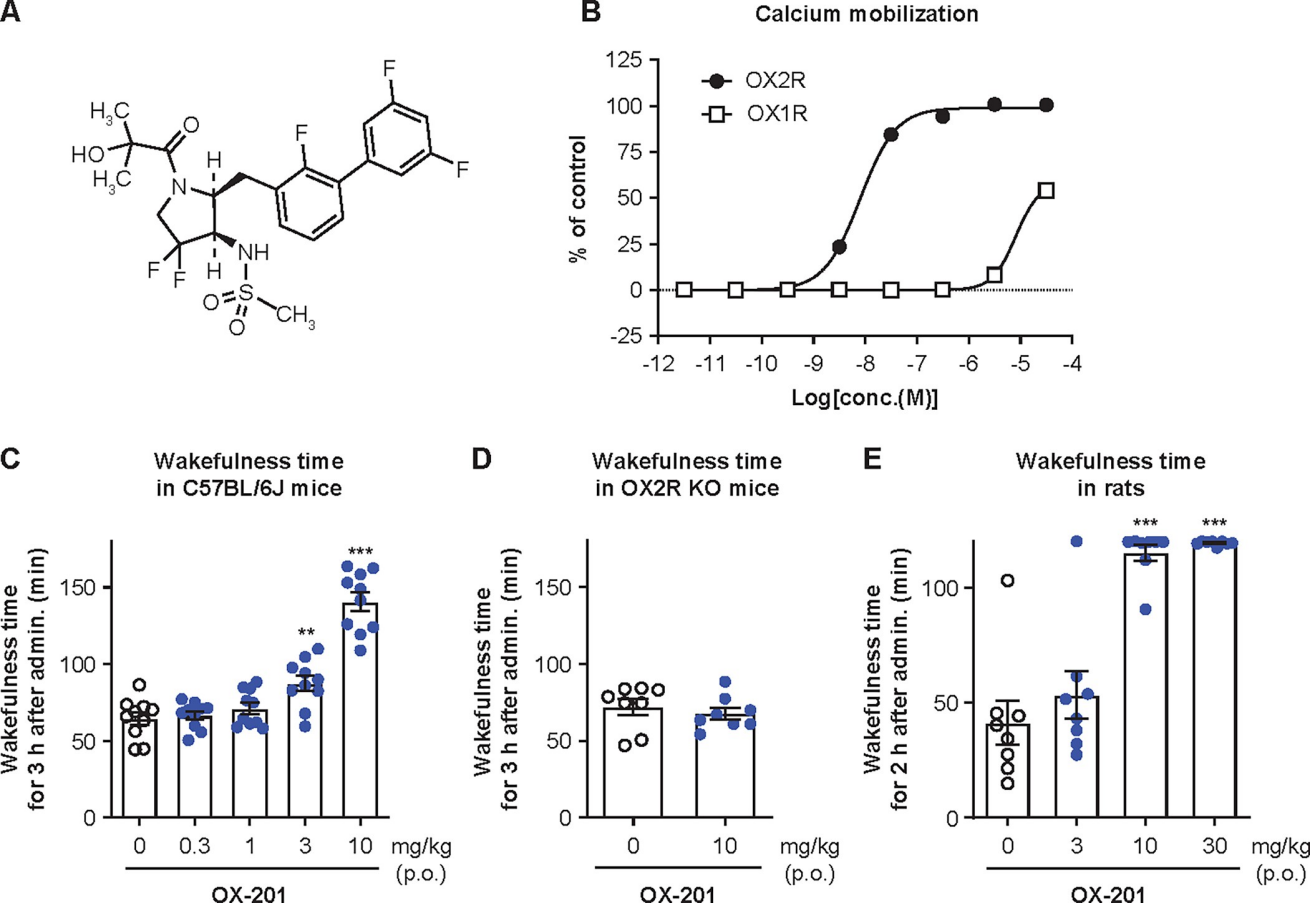
OX-201 selectively activated OX2R and produced wake-promoting effects via OX2R activation in mice and rats. (A) Chemical structure of OX-201. (B) Effects of OX-201 on calcium mobilization in hOX2R/CHO-K1 cells and hOX1R/CHO-K1 cells. The responses to 0.5% DMSO in the absence and presence of 100 nM OX-A (positive control) were used to represent the 0% and 100% responses, respectively. n = 4. (C) Effects of oral (p.o.) administration of OX-201 on wakefulness time in C57BL/6J mice. n = 10, **P = 0.003, ***P < 0.001 versus vehicle-treated mice. (D) Effects of OX-201 (p.o.) on wakefulness time in OX2R KO mice. n = 8. (E) Effects of OX-201 (p.o.) on wakefulness time in rats. n = 8, ***P < 0.001 versus vehicle-treated rats. Data are presented as mean ± standard error of the mean.

The chemical structure of OX-201 is shown in Fig 1A. OX-201 showed potent OX2R agonistic activity, with an EC_50_ of 8.0 nM in a calcium mobilization assay in hOX2R/CHO-K1 cells, and showed very weak OX1R agonistic activity, with an EC_50_ of 8.1 μM in hOX1R/CHO-K1 cells (Fig 1B). Thus, OX-201 had 1000-fold selectivity for OX2R over OX1R. *In vitro* assays for off-target profiling revealed that OX-201 at 10 μM did not show >50% inhibition or stimulation for 102 tested enzymes, receptors, or ion channels, except cannabinoid CB1 receptor (51% inhibition) and progesterone receptor B (53% inhibition) (S2 and S3 Tables), indicating it has minimal off-target activity. These results suggest that OX-201 is a potent and highly selective OX2R agonist.

Oral administration of OX-201 showed a pharmacokinetic profile suitable for *in vivo* studies in C57Bl/6J mice (T_max_ approximately 1 h; MRT approximately 5 h) (S1 Fig and S4 Table). Accordingly, efficacy of OX-201 was evaluated during 3 h after oral administration in mice. Humans are diurnal primates with monophasic wakefulness/sleep structure, while rodents are nocturnal animals with polyphasic wakefulness/sleep structure. Therefore, OX-201 was administered at ZT5 (sleep phase) when activity of orexin neurons is low. As a result, OX-201 at 3 and 10 mg/kg significantly increased wakefulness time in EEG/EMG recordings during the sleep phase in C57BL/6J mice (P = 0.003 and P < 0.001, respectively; Fig 1C). Under similar experimental conditions, OX-201 at 10 mg/kg did not increase wakefulness time in OX2R KO mice (Fig 1D). These results suggest OX-201 produces wake-promoting effects via OX2R activation in mice.

In rats, oral administration of OX-201 showed a pharmacokinetic profile suitable for *in vivo* studies (T_max_ approximately 1.3–1.8 h; MRT approximately 2–6 h) (S2A Fig and S5 Table). During 2 h after oral administration at ZT5, OX-201 at 10 and 30 mg/kg significantly increased wakefulness time during the sleep phase in rats (P < 0.001; Fig 1E). These results were used to guide the selection of OX-201 doses in other studies reported here.

#### Effects of danavorexton and OX-201 on the activity of inspiratory neurons in the pre-Bötzinger complex in rat medullary slices

**Fig 2:**
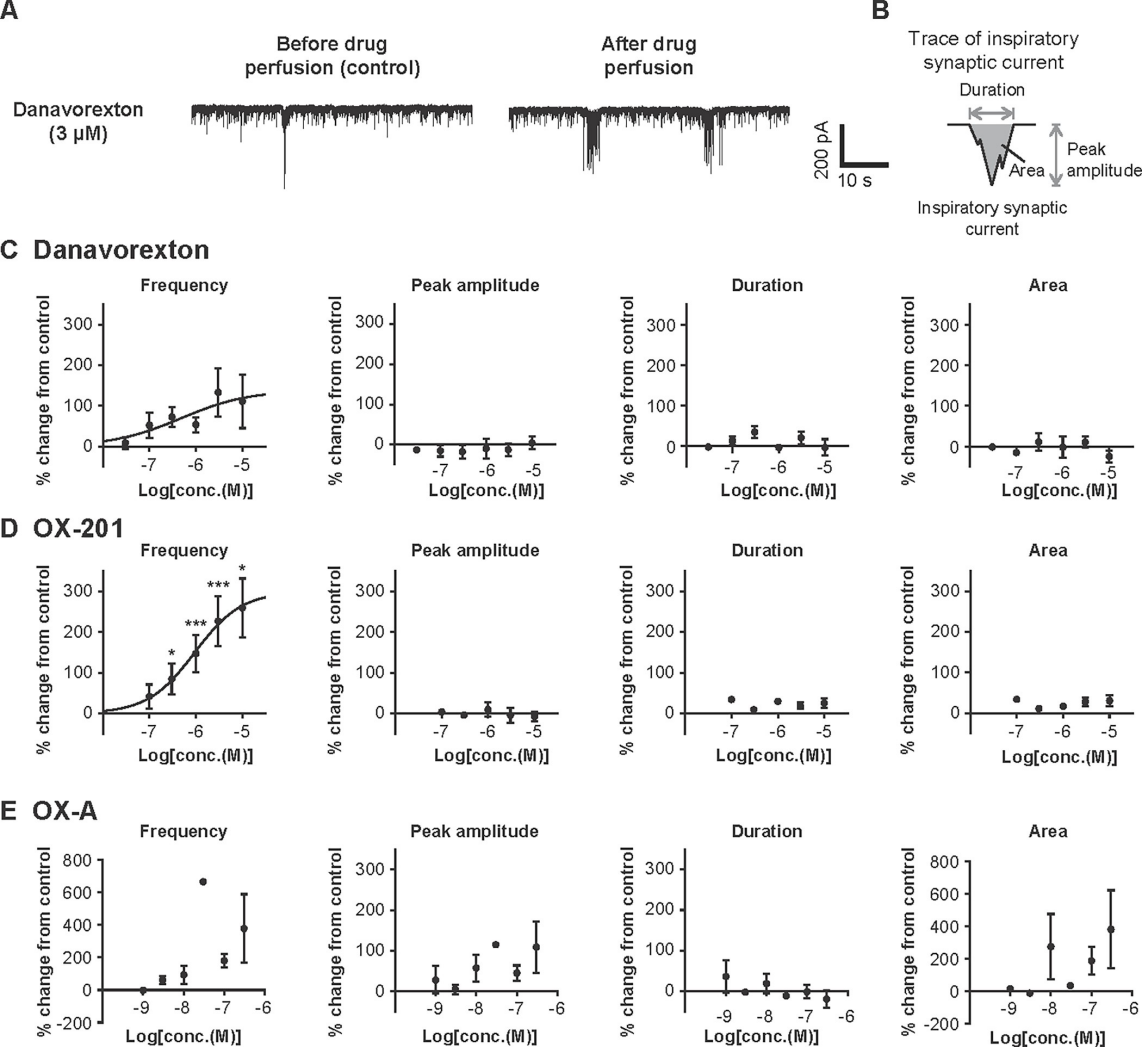
Danavorexton, OX-201, and OX-A activated inspiratory neurons in the pre-Bötzinger complex in rat medullary slices. (A) Representative traces by whole-cell patch clamp recording of inspiratory neurons in the pre-Bötzinger complex before and after perfusion with danavorexton at 3 μM. (B) The method to calculate peak amplitude, duration, and area of inspiratory synaptic currents from recorded signals. (C) Effects of danavorexton on the frequency, peak amplitude, duration, and area of inspiratory synaptic currents. Numbers of neurons recorded in each concentration (n) are 4 at 30 nM, 4 at 100 nM, 5 at 300 nM, 5 at 1 μM, 4 at 3 μM, and 4 at 10 μM (frequency); 4 at 30 nM, 3 at 100 nM, 5 at 300 nM, 3 at 1 μM, 4 at 3 μM, and 3 at 10 μM (peak amplitude and area); 4 at 30 nM, 3 at 100 nM, 5 at 300 nM, 4 at 1 μM, 4 at 3 μM, and 3 at 10 μM (duration). (D) Effects of OX-201 on the frequency, peak amplitude, duration, and area of inspiratory synaptic currents. Numbers of neurons recorded in each concentration (n) are 3 at 100 nM, 10 at 300 nM, 12 at 1 μM, 10 at 3 μM, and 6 at 10 μM. (E) Effects of OX-A on the frequency, peak amplitude, duration, and area of inspiratory synaptic currents. Numbers of neurons recorded in each concentration (n) are 4 at 1 nM, 3 at 3 nM, 6 at 10 nM, 1 at 30 nM, 7 at 100 nM, and 3 at 300 nM. Neuronal activity was recorded in the presence of vehicle followed by the application of drugs. Mean values in the frequency, peak amplitude, duration, and area of inspiratory synaptic currents during the last 1–2 min in the presence of vehicle were used as control values, and those during the last 1–2 min with stimulation by each concentration of drug were used to calculate percent changes from control values. *P < 0.05, ***P < 0.001 versus control. Data are presented as mean ± standard error of the mean.

To examine the effects of danavorexton, OX-201, and OX-A on the activity of inspiratory neurons in the pre-Bötzinger complex, whole-cell patch clamp recording using rat medullary slices was conducted. As shown by representative traces, danavorexton appeared to increase the firing of inspiratory neurons in the pre-Bötzinger complex (Fig 2A). Detailed assessment of each inspiratory synaptic current to measure frequency, peak amplitude, duration, and area was conducted (Fig 2B). Although not statistically significant, danavorexton showed a dose-dependent increase in the frequency of inspiratory synaptic currents (EC_50_ 470 nM) (Fig 2C). OX-201 significantly and dose-dependently increased the frequency of inspiratory synaptic currents (EC_50_ 940 nM) (Fig 2D); the effect of OX-201 at 0.3, 1, 3, and 10 μM reached statistical significance (P = 0.027, P < 0.001, P < 0.001, and P = 0.025, respectively). OX-A also showed a tendency to increase the frequency of inspiratory synaptic currents (Fig 2E). The lack of statistical significance in increasing the frequency of inspiratory synaptic currents by both danavorexton and OX-A may be due to the smaller number of neurons used for their characterization (mean number of neurons used at one concentration: 4.3 for danavorexton, 8.2 for OX-201, and 4 for OX-A). Different from danavorexton and OX-201, OX-A appeared to increase peak amplitude and area (Fig 2E). Danavorexton, OX-201, and OX-A did not affect duration at concentrations used in this study (Fig 2C–2E). Overall, these results suggest that OX2R-selective agonists increase the activity of inspiratory neurons in the pre-Bötzinger complex in rat medullary slices.

#### Effects of danavorexton and OX-201 on burst activity recorded from the cervical ventral root in rat isolated brainstem–spinal cord preparations

**Fig 3:**
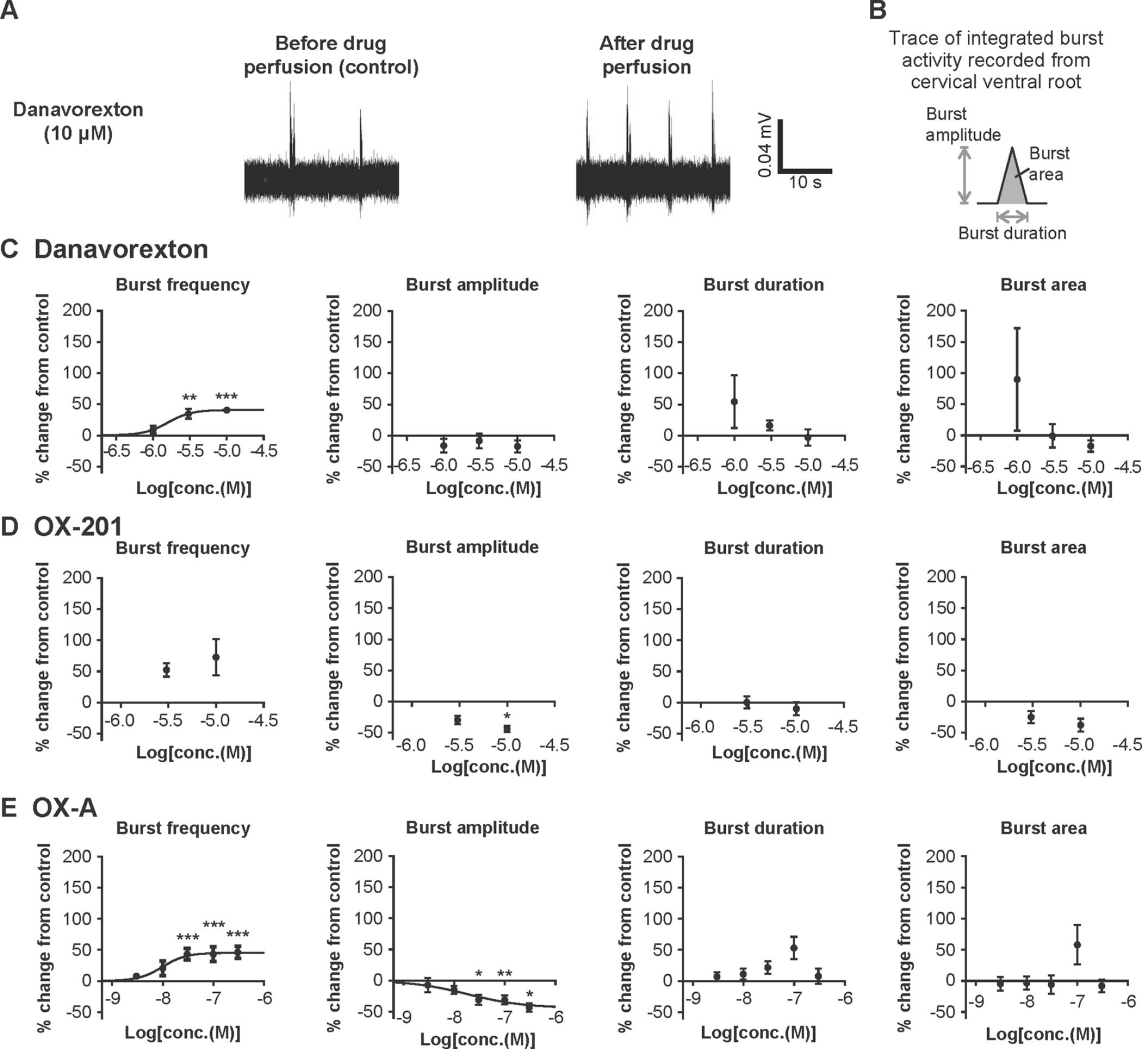
Danavorexton, OX-201, and OX-A increased burst activity in rat isolated brainstem−spinal cord preparations. (A) Representative bandpass-filtered (0.01–3000 Hz) electrophysiological traces of burst activity recorded from the cervical (C3–C5) ventral root before and after perfusion with danavorexton at 10 μM. (B) The method to calculate burst amplitude, burst duration, and burst area from integrated signals. (C) Effects of danavorexton on burst frequency, burst amplitude, burst duration, and burst area. Numbers of tissues recorded in each concentration (n) are 5 at 1 μM, 5 at 3 μM, and 6 at 10 μM. (D) Effects of OX-201 on burst frequency, burst amplitude, burst duration, and burst area. Curve fitting was not implemented because only two concentrations of OX-201 were tested. Numbers of tissues recorded in each concentration (n) are 5 at 3 μM and 4 at 10 μM. (E) Effects of OX-A on burst frequency, burst amplitude, burst duration, and burst area. Numbers of tissues recorded in each concentration (n) are 6 at 3 nM, 6 at 10 nM, 8 at 30 nM, 14 at 100 nM, and 6 at 300 nM. Burst activity was recorded in the presence of vehicle followed by the application of drugs. Mean values in burst frequency, burst amplitude, burst duration, and burst area during the last 2 min in the presence of vehicle were used as control values, and those during the last 2 min of drug perfusion were used to calculate percent changes from control values. *P < 0.05, **P < 0.01, and ***P < 0.001 versus control. Data are presented as mean ± standard error of the mean.

To examine the effects of danavorexton, OX-201, and OX-A on the burst activity in the cervical ventral root, which contains phrenic motoneuron axons, electrophysiological recording from C3–C5 ventral root using rat isolated brainstem–spinal cord preparations was conducted. As shown by representative bandpass-filtered (0.01–3000 Hz) traces, danavorexton appeared to increase burst activity in the cervical ventral root (Fig 3A). Detailed assessment of each burst to measure burst frequency, burst amplitude, burst duration, and burst area was conducted (Fig 3B). Danavorexton and OX-A significantly and dose-dependently increased burst frequency (EC_50_ 1.6 μM and 9.9 nM, respectively) (Fig 3C and 3E). The effect of danavorexton at 3 and 10 μM and OX-A at 30, 100, and 300 nM reached statistical significance (danavorexton: P = 0.0046 at 3 μM and P < 0.001 at 10 μM; OX-A: all P < 0.001). Although not significant and likely due to the smaller number of neurons used for characterization, OX-201 tended to increase burst frequency (Fig 3D); the mean number of neurons used at one concentration was 5.3 for danavorexton, 4.5 for OX-201, and 8 for OX-A, respectively. OX-201 and OX-A, but not danavorexton, significantly decreased burst amplitude (EC_50_ not determined and 19 nM, respectively) (Fig 3C–3E). The effect of OX-201 at 10 μM and OX-A at 30, 100, and 300 nM reached statistical significance (OX-201: P = 0.036; OX-A: P = 0.030 at 30 nM, P = 0.0023 at 100 nM, and P = 0.016 at 300 nM). Danavorexton, OX-201, and OX-A did not affect burst duration and burst area in this study (Fig 3C, 3D, and 3E). Overall, these results suggest that OX2R-selective agonists increase burst activity, mainly by increasing burst frequency, in the cervical ventral root in rat isolated brainstem–spinal cord preparations and accordingly, OX2R agonists may activate phrenic motoneurons.

#### Effects of danavorexton and OX-201 on hypoglossal motoneuron activity in rat medullary slices

**Fig 4:**
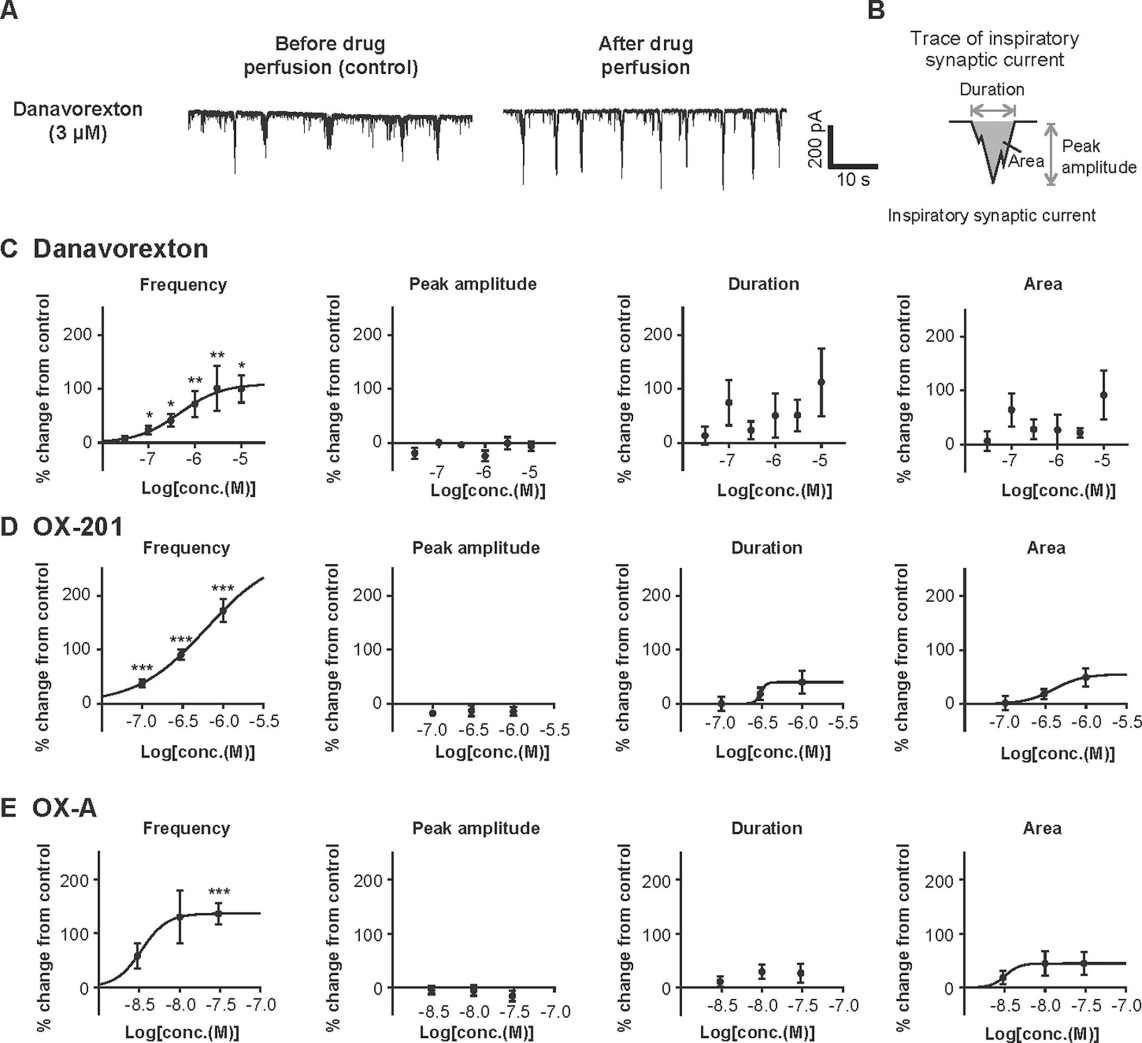
Danavorexton, OX-201, and OX-A activated hypoglossal motoneurons in rat medullary slices. (A) Representative traces by whole-cell patch clamp recording of hypoglossal motoneurons before and after perfusion with danavorexton at 10 μM. (B) The method to calculate peak amplitude, duration, and area of inspiratory synaptic currents from recorded signals. (C) Effects of danavorexton on the frequency, peak amplitude, duration, and area of inspiratory synaptic currents. Numbers of neurons recorded in each concentration (n) are 5 at 30 nM, 7 at 100 nM, 7 at 300 nM, 8 at 1 μM, 5 at 3 μM, and 5 at 10 μM. (D) Effects of OX-201 on the frequency, peak amplitude, duration, and area of inspiratory synaptic currents. Numbers of neurons recorded in each concentration (n) are 10 at 100 nM, 11 at 300 nM, and 10 at 1 μM. (E) Effects of OX-A on the frequency, peak amplitude, duration, and area of inspiratory synaptic currents. Numbers of neurons recorded in each concentration (n) are 10 at 3 nM, 10 at 10 nM, and 8 at 30 nM. Neuronal activity was recorded in the presence of vehicle followed by the application of drugs. Mean values in the frequency, peak amplitude, duration, and area of inspiratory synaptic currents during the last 1–2 min in the presence of vehicle were used as control values, and those during the last 1–2 min with stimulation by each concentration of drugs were used to calculate percent changes from control values. *P < 0.05, **P < 0.01, ***P < 0.001 versus control. Data are presented as mean ± standard error of the mean.

To examine the effects of danavorexton, OX-201, and OX-A on the activity of hypoglossal motoneurons, whole-cell patch clamp recording using rat medullary slices was conducted. As shown by representative traces, danavorexton appeared to increase the firing of hypoglossal motoneurons (Fig 4A). Detailed assessment of each inspiratory synaptic current to measure the frequency, peak amplitude, duration, and area was conducted (Fig 4B). Danavorexton, OX-201, and OX-A significantly and dose-dependently increased the frequency of inspiratory synaptic currents (EC_50_ 470, 620, and 3.4 nM, respectively) (Fig 4C–4E). The effect of danavorexton at 0.1, 0.3, 1, 3, and 10 μM, OX-201 at 0.1, 0.3, and 1 μM, and OX-A at 30 nM reached statistical significance (danavorexton: P = 0.044 at 0.1 μM, P = 0.011 at 0.3 μM, P = 0.0075 at 1 μM, P = 0.0014 at 3 μM, and P = 0.047 at 10 μM; OX-201: all P < 0.001; OX-A: P < 0.001). Danavorexton and OX-201 showed tendency to increase the duration of inspiratory synaptic currents (Fig 4C and 4D). Danavorexton, OX-201, and OX-A showed tendency to increase the area of inspiratory synaptic currents (Fig 4C–4E). Danavorexton, OX-201, and OX-A did not affect the peak amplitude in this study (Fig 4C–4E). To understand the conclusions in these parameters with lower magnitude of percent change, a higher number of neurons need to be assessed; mean number of neurons used at one concentration was 6.2 for danavorexton, 10.3 for OX-201, and 9.3 for OX-A, respectively. Overall, these results suggest that OX2R-selective agonists increase the activity of hypoglossal motoneurons in rat medullary slices.

#### Effects of OX-201 on the activity of the diaphragm in EMG recordings in anesthetized rats

**Fig 5:**
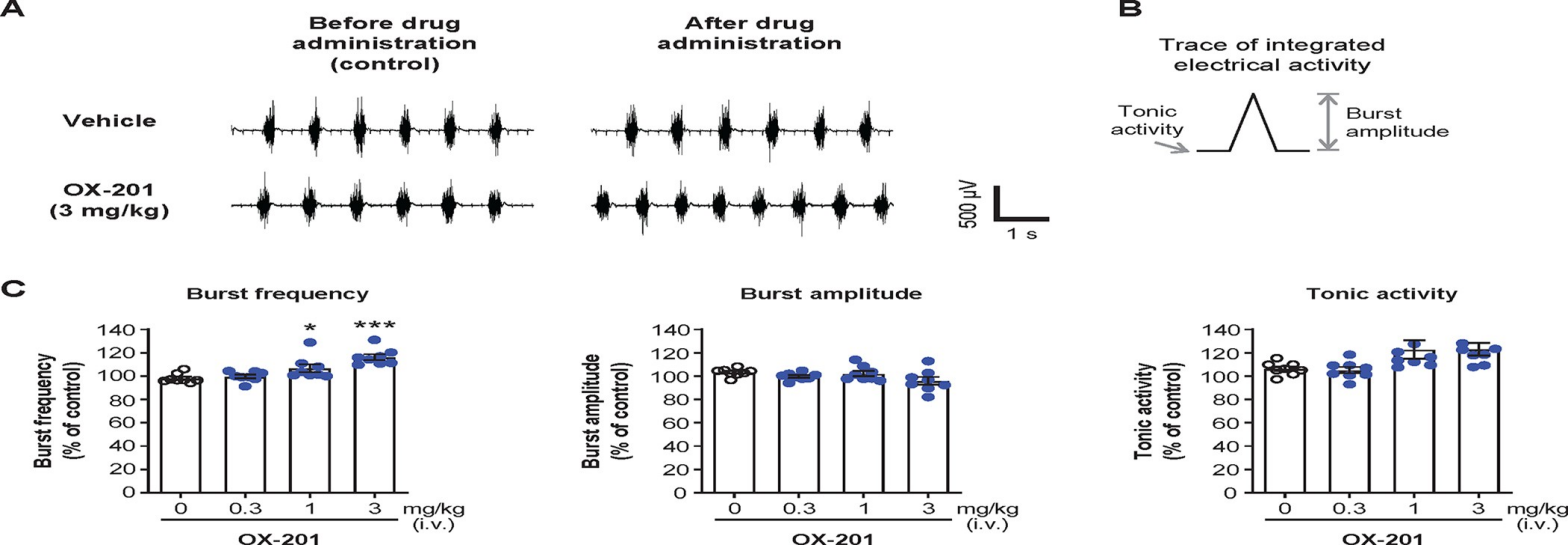
Intravenous (i.v.) OX-201 administration significantly increased burst frequency of the diaphragm in anesthetized rats. (A) Representative traces by EMG recording of the diaphragm before and after administration of vehicle or OX-201 at 3 mg/kg. (B) The method to calculate burst amplitude and tonic activity from integrated EMG signals. (C) Effects of OX-201 on burst frequency, burst amplitude, and tonic activity of the diaphragm. Tonic activity was defined as the mean value of integrated EMG signals between bursts. Mean values in burst frequency, burst amplitude, and tonic activity during 2 min before vehicle or OX-201 administration were used as control values, and those during 10 min after vehicle or OX-201 administration were used to calculate percent changes from control values. n = 8, *P = 0.040, ***P < 0.001 versus vehicle-treated rats. Data are presented as mean ± standard error of the mean.

Due to the technical feasibility of the experiment, anesthetized rats were used for EMG recordings of the diaphragm. OX-201 was administered intravenously to anesthetized rats in this experiment. To understand the relationship between the plasma effective concentration of OX-201 for arousal and for activation of the diaphragm, plasma concentration of OX-201 after intravenous administration was measured. The maximum plasma concentrations of OX-201 after intravenous administration at 0.3 and 1 mg/kg were similar to those after oral administration at 3 and 10 mg/kg, respectively (S2 Fig and S5 and S6 Tables). Because oral administration of OX-201 at ≥10 mg/kg produced potent arousal effects in rats (Fig 1E), intravenous administration of OX-201 at 0.3, 1, and 3 mg/kg was used to assess its potential to activate the diaphragm in EMG recordings in anesthetized rats. As shown by representative traces, intravenous administration of OX-201 at 3 mg/kg appeared to increase the activity of the diaphragm (Fig 5A). Detailed assessment of each burst to measure burst frequency, burst amplitude, and tonic activity were conducted (Fig 5B). Burst amplitude indicates the peak diaphragm activity during burst, and tonic activity indicates the basal diaphragm activity between bursts. Intravenous administration of OX-201 at 1 and 3 mg/kg significantly increased burst frequency of the diaphragm (P = 0.040 and P < 0.001, respectively; Fig 5C). Notably, plasma exposure of OX-201 after intravenous administration at 1 mg/kg is comparable with exposure after oral administration at 10 mg/kg, with a potent wake-promoting effect in rats (Fig 1E). Alternatively, OX-201 did not show any significant effects in burst amplitude or tonic activity at dosages used in this study (Fig 5C). These results suggest OX-201 increases diaphragm activity in anesthetized rats.

### Effects of OX-201 on genioglossus muscle activity in EMG recordings in anesthetized rats

**Fig 6:**
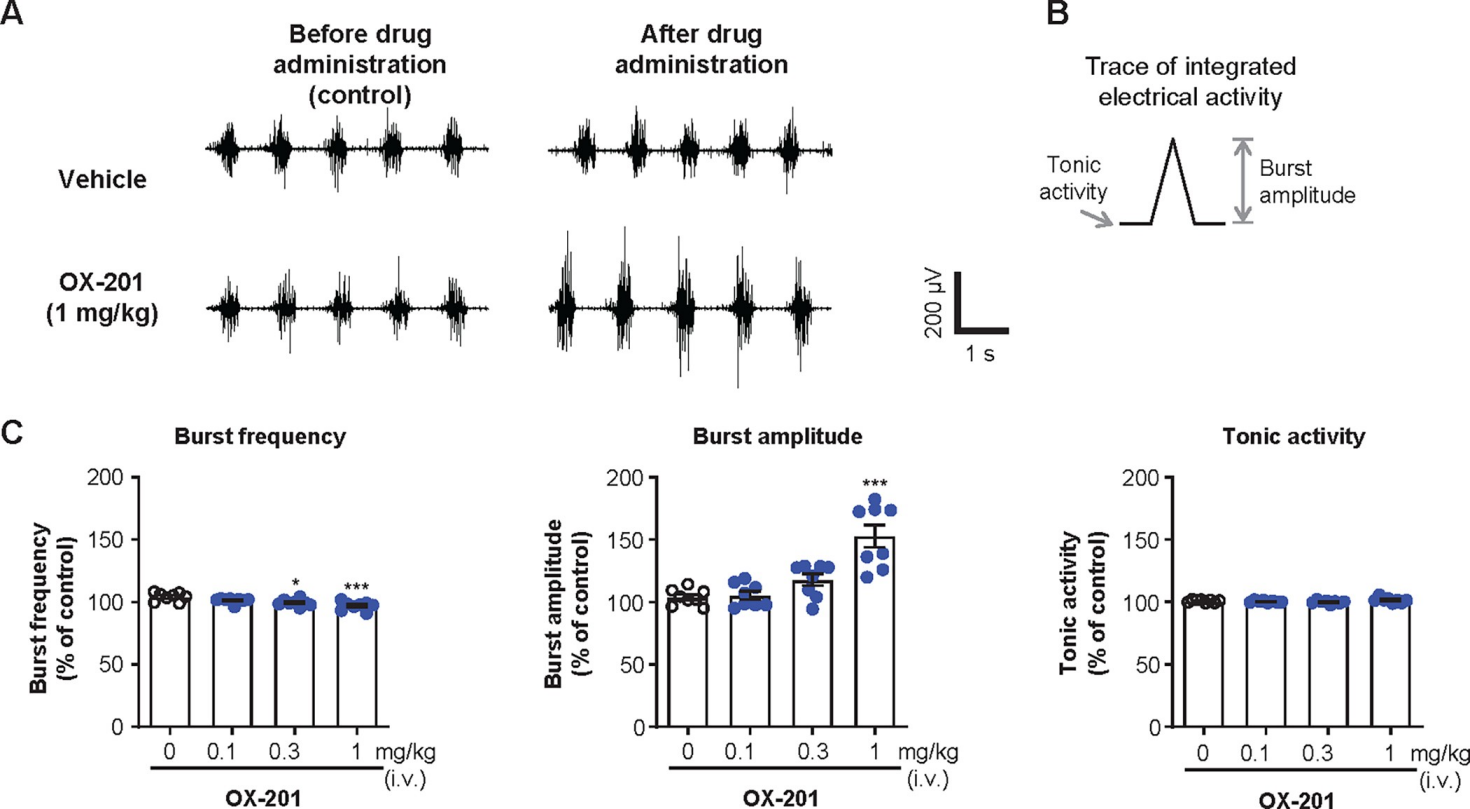
Intravenous (i.v.) OX-201 administration significantly increased burst amplitude of the genioglossus muscle in anesthetized rats. (A) Representative traces by EMG recordings of the genioglossus muscle before and after administration of vehicle or OX-201 at 1 mg/kg. (B) The method to calculate burst amplitude and tonic activity from integrated EMG signals. Tonic activity was defined as the mean value of integrated EMG signals between bursts. (C) Effects of OX-201 on burst frequency, burst amplitude, and tonic activity of the genioglossus muscle. Mean values in burst frequency, burst amplitude, and tonic activity during 2 min before vehicle or OX-201 administration were used as control values, and those during 10 min after vehicle or OX-201 administration were used to calculate percent changes from control values. n = 8, *P = 0.011, ***P < 0.001 versus vehicle-treated rats. Data are presented as mean ± standard error of the mean.

Effects of intravenous administration of OX-201 on genioglossus muscle activity in anesthetized rats were examined by EMG recording. As shown by representative traces, OX-201 appeared to increase genioglossus muscle activity (Fig 6A). Detailed assessments of each burst to measure burst frequency, burst amplitude, and tonic activity were conducted (Fig 6B). Burst amplitude indicates the peak activity during the burst; tonic activity indicates the basal activity between bursts. Intravenous administration of OX-201 at 0.3 and 1 mg/kg significantly decreased burst frequency of the genioglossus muscle (P = 0.011 and P < 0.001, respectively; Fig 6C), and at 1 mg/kg significantly increased burst amplitude (P < 0.001; Fig 6C). OX-201 did not show any significant effects in tonic activity at dosages used in this study (Fig 6C). These results suggest OX-201 increases genioglossus muscle activity, especially burst amplitude, in anesthetized rats.

### Effects of OX-201 on respiratory activity measured by whole-body plethysmography in free-moving mice

**Fig 7:**
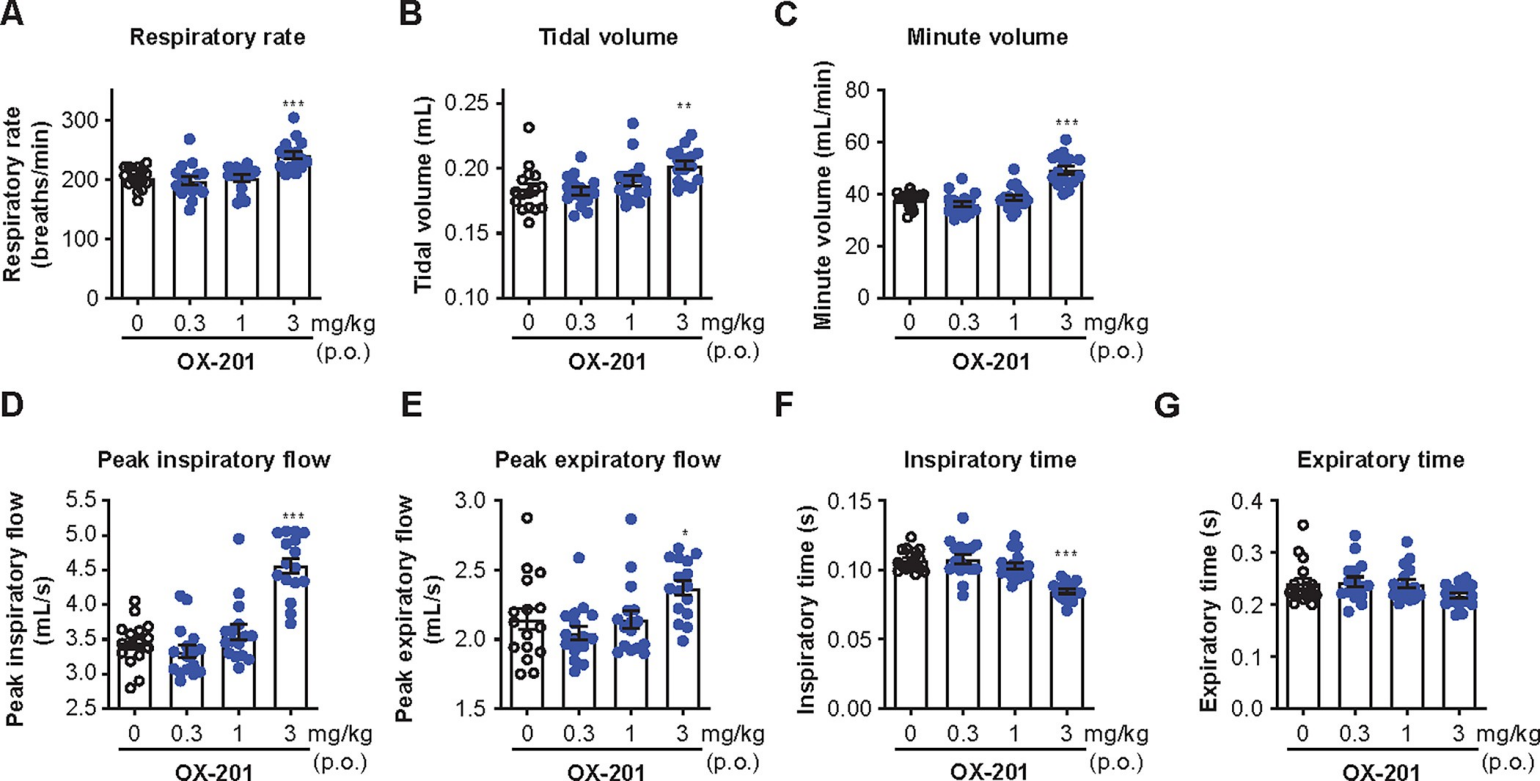
Oral (p.o.) administration of OX-201 significantly increased respiratory activity in free-moving mice. Effects of OX-201 on the (A) respiratory rate, (B) tidal volume, (C) minute volume, (D) peak inspiratory flow, (E) peak expiratory flow, (F) inspiratory time, and (G) expiratory time were measured. The mean values in each parameter during 3 h after vehicle or OX-201 administration were calculated. n = 16, *P = 0.035, **P = 0.0017, ***P < 0.001 versus vehicle-treated mice. Data are presented as mean ± standard error of the mean.

Finally, to examine the effects of OX-201 on respiratory activity, whole-body plethysmography recording was conducted in free-moving mice. Oral administration of OX-201 (3 mg/kg) at ZT5 during the sleep phase significantly increased respiratory rate (P < 0.001), tidal volume (P = 0.0017), minute volume (P < 0.001), peak inspiratory flow (P < 0.001), and peak expiratory flow (P = 0.035), and decreased inspiratory time (P < 0.001) during 3 h after drug administration (Fig 7A–7F). Although OX-201 showed similar trends in both inspiratory and expiratory time, the effects of OX-201 on expiratory time did not reach statistical significance at up to 3 mg/kg in this study (Fig 7F and 7G). These results suggest OX-201 increases respiratory activity in free-moving mice.

## Discussion

Inspiratory neurons in the pre-Bötzinger complex located in the ventrolateral medulla are critical for the generation of inspiratory rhythm. Additionally, they regulate activity of the diaphragm and genioglossus muscle through controlling the activity of phrenic motoneurons in the cervical spinal cord and hypoglossal motoneurons in the dorsomedial medulla, respectively [3]. Inspiratory neurons in the pre-Bötzinger complex are known to show rhythmic firing in phase with inspiratory bursts of the hypoglossal nerve [41–43]. In this study, however, we could not assess synchronization between neuronal activity in the pre-Bötzinger complex and bursts of the hypoglossal nerve. However, the frequency of rhythmic firing of possible inspiratory neurons in the pre-Bötzinger complex and hypoglossal motoneurons was similar and align with previously reported results [42, 43]. Therefore, we believe that we examined inspiratory neurons in the pre-Bötzinger complex.

Previous studies report that OX-A and OX-B can activate inspiratory neurons in the pre-Bötzinger complex, phrenic motoneurons, and hypoglossal motoneurons [19, 24, 25]. In line with these reports, in this study, OX-A showed a tendency to activate inspiratory neurons in the pre-Bötzinger complex and activated hypoglossal motoneurons in rat medullary slices, and increased burst activity in the cervical ventral root in rat isolated brainstem–spinal cord preparations.

Danavorexton and OX-201 showed a tendency and significant effect, respectively, in increasing the activity of inspiratory neurons in the pre-Bötzinger complex and these OX2R-selective agonists significantly increased the activity of hypoglossal motoneurons in rat medullary slices. OX2R protein is expressed in the pre-Bötzinger complex in rodents [46, 47]. Therefore, it is conceivable that OX2R agonists directly stimulate inspiratory neurons in the pre-Bötzinger complex. However, because the connection between inspiratory neurons in the pre-Bötzinger complex and hypoglossal motoneurons may be preserved in this preparation, we could not determine whether OX2R agonists directly and/or indirectly activated hypoglossal motoneurons from this study. Furthermore, danavorexton and OX-201 showed a significant effect and a tendency, respectively, in increasing burst activity recorded from the cervical ventral root, which contains phrenic motoneuron axons, mainly by increasing burst frequency, in rat isolated brainstem–spinal cord preparations. Accordingly, danavorexton and OX-201 may activate phrenic motoneurons. Again, because the connection between inspiratory neurons in the pre-Bötzinger complex and phrenic motoneurons may be preserved in this preparation, it was unclear from this study whether OX2R agonists directly and/or indirectly activated phrenic motoneurons. Neurons in the parafacial respiratory group, which is associated with respiratory rhythm generation [48], are reported to express OX2R protein in mice [47]. The Kölliker-Fuse nucleus, in the dorsolateral pons, may also be involved in the OX2R agonist–induced modulation of respiratory function [49]. More detailed functional studies, including neurons in the parafacial respiratory group and the Kölliker-Fuse nucleus, would be needed to clarify target sites/neurons for respiratory regulation by OX2R-selective agonists.

OX-201 increased the respiratory rate of free-moving mice in whole-body plethysmography analysis. This result was in line with OX-201–induced increases in the frequency of inspiratory synaptic currents of inspiratory neurons in the pre-Bötzinger complex and hypoglossal motoneurons, and burst frequency recorded from the cervical ventral root. Because the diaphragm and genioglossus muscle produce spontaneous bursts during inspiration, burst frequency of these muscles would be related to respiratory rate of these mice. Thus, we expected to see increased burst frequency in both the diaphragm and genioglossus muscle. However, *in vivo* studies revealed that OX-201 increased burst frequency of the diaphragm, whereas it mainly increased burst amplitude of the genioglossus muscle in anesthetized rats; OX-201 slightly reduced burst frequency of the genioglossus muscle. Although underlying mechanisms of action are unclear, the impact of anesthetic drugs used in these studies needs to be considered. In the EMG recording of the diaphragm, isoflurane was used, whereas in the EMG recording of the genioglossus muscle, urethane was used based on the previous report [25]. Isoflurane and urethane may have different impacts on neurons involved in respiratory rhythm regulation, resulting in a discrepancy between *in vitro* electrophysiological analysis, whole-body plethysmography analysis in free-moving mice, and EMG recording of the diaphragm and genioglossus muscle under different anesthetics. Ideally, diaphragm and genioglossus muscle activity should be simultaneously measured under one anesthetic agent. By considering the increased burst frequency of the diaphragm, we attempted to simultaneously measure diaphragm and genioglossus muscle activity under isoflurane anesthesia. However, we could not conduct this experiment due to technical obstacles, which is a recognized limitation of this study. Such simultaneous recording may be technically possible under urethane anesthesia; thus, it would be desirable to perform the study in future investigations. Although further assessments are needed, our results suggest that OX2R agonists can modulate respiratory functions by increasing diaphragm and genioglossus muscle activity through stimulation of the neuronal network including neurons in the pre-Bötzinger complex, and phrenic and hypoglossal motoneurons in rodents.

Respiratory activity is closely associated with sleep/wake states in rodents. For example, tidal volume was higher during wake than in sleep in both C57BL/6J mice and rats [50, 51]. Thus, OX-201–induced activation of respiratory functions could be secondary to increased wakefulness. However, our study for assessing diaphragm and genioglossus muscle activity was conducted under anesthetized conditions. With modulation of the neuronal circuit associated with respiration in *in vitro* studies, we believe OX2R agonists have direct effects on respiration.

After surgery, delayed emergence from anesthesia is related to various complications, including respiratory/cardiovascular dysfunction, delirium, and cognitive decline [52–54]. Use of opioids to manage pain are also associated with severe adverse events, including sedation and respiratory depression [7]. Thus, drugs that facilitate recovery from anesthesia/opioid-induced sedation and suppress OIRD without compromising analgesia are required [9]. Recent studies indicate that stimulation of the sleep/wake system in the brain, such as the orexin system, can facilitate emergence from anesthesia [55]. Analgesic effects of orexins have been suggested in animal models [56]; importantly there is no evidence that an OX2R agonist can cancel an opioid’s analgesic effects. We recently observed that danavorexton promotes recovery from anesthesia/fentanyl-induced sedation and inhibits OIRD without compromising fentanyl analgesia in pre-clinical studies using rats and monkeys [57]. Further studies are needed to clinically characterize danavorexton.

CSA is associated with blocked or weakened respiratory signal from the brainstem respiratory center to the diaphragm [6]; thus, a drug that can activate the diaphragm may have therapeutic potential for CSA. OSA is associated with the obstruction of the upper airway [6]; thus, a drug that can increase genioglossus muscle activity may have therapeutic efficacy for OSA. For example, phenylephrine, an α_1_ adrenergic receptor agonist, and scopolamine, a muscarine antagonist, increased genioglossus muscle activity in rats [58, 59]. AD109, a combination of atomoxetine, a noradrenaline reuptake inhibitor, and R form of oxybutynin, a muscarine antagonist, increased genioglossus muscle activity and decreased Apnea–Hypopnea Index score, a validated measure of severity of sleep apnea, in individuals with OSA [13]. Dronabinol, a CB1 antagonist, increased genioglossus muscle activity in rats [60], and decreased Apnea–Hypopnea Index score in patients with OSA [12]. Because OX2R agonists demonstrate activation of the diaphragm and genioglossus muscle, they may have therapeutic potential for both CSA and OSA.

Because OX2R agonists have potent arousal effects, their application to improve respiratory function during the sleep phase, such as enhancement of respiratory function in patients with sleep apnea, should be carefully considered. Oral administration of 10 mg/kg OX-201 in rats significantly increased wakefulness time in the sleep phase, and intravenous administration of 1 mg/kg OX-201 activated the diaphragm and genioglossus muscle in anesthetized rats. The C_max_ of OX-201 after oral administration at 10 mg/kg and after intravenous administration at 1 mg/kg were similar. Thus, the effective concentration for wakefulness and for respiration could be similar in rodents. However, anesthetized conditions may impact effective concentrations of OX2R agonists for activation of the diaphragm and genioglossus muscle. Moreover, humans have monophasic sleep/wake structure, whereas rodents have polyphasic sleep/wake structure. Therefore, effective concentrations for arousal and respiratory regulation by OX2R agonists could be different between rodents and humans. Further pre-clinical and clinical studies are needed.

## Conclusion

OX2R agonists can modulate respiratory function by increasing diaphragm and genioglossus muscle activity through stimulation of the neuronal network including inspiratory neurons in the pre-Bötzinger complex, and phrenic and hypoglossal motoneurons in rodents. If confirmed in humans, OX2R agonists may present therapeutic effects in clinical conditions in which respiratory functioning is compromised. Clinical studies of danavorexton to investigate its efficacy in patients with OSA, as well as post-anesthesia recovery, have recently completed (ClinicalTrials.gov NCT05180890 and NCT05025397).

## Supporting information

S1 Fig Plasma concentrations of OX-201 after oral (p.o.) administration in C57BL/6J mice. Time-dependent changes in plasma concentrations of OX-201 (p.o.) in C57BL/6J mice. n = 3, mean ± standard error of the mean.
(TIF)

S2 Fig Plasma concentrations of OX-201 after administration in rats. (a) Time-dependent changes in plasma concentrations of OX-201 after oral (p.o.) administration in rats. n = 3–4, mean ± standard error of the mean (SEM). (b) Time-dependent changes in plasma concentrations of OX-201 after intravenous (i.v.) administration in rats. n = 5, mean ± SEM.
(TIF)

S1 Table Effects of 0.1% DMSO on the frequency of inspiratory synaptic currents of inspiratory neurons in the pre-Bötzinger complex and hypoglossal motoneurons, and burst frequency recorded from the cervical ventral root. Neuronal activity or burst activity recorded from the cervical ventral root under basal conditions was recorded for 2–20 min, followed by recording in the presence of 0.1% DMSO. DMSO was perfused for 30 min (inspiratory neurons in the pre-Bötzinger complex and hypoglossal motoneurons) or 30–40 min (cervical ventral root). Mean values in the frequency during the last 1–2 min (inspiratory neurons in the pre-Bötzinger complex and hypoglossal motoneurons) or burst frequency during the last 2 min (cervical ventral root) of basal conditions and those during the last 1–2 min (inspiratory neurons in the pre-Bötzinger complex and hypoglossal motoneurons) or 2 min (cervical ventral root) of 0.1% DMSO perfusion were compared. Numbers of recorded neurons or tissues (n) are 8 (inspiratory neurons in the pre-Bötzinger complex), 5 (hypoglossal motoneurons), and 15 (cervical ventral root). Data are presented as mean ± standard error of the mean. DMSO, dimethyl sulfoxide.
(PDF)

S2 Table Percent inhibition of various enzymes by OX-201 at 10 μM. ATP, adenosine triphosphate; EGF, epidermal growth factor; HMG-CoA, 3-hydroxy-3-methyl-glutaryl coenzyme A; ROCK1, rho-associated, coiled-coil-containing protein kinase 1.
(PDF)

S3 Table Percent inhibition of various receptors or ion channels by OX-201 at 10 μM. AMPA, α-amino-3-hydroxy-5-methyl-4-isoxazolepropionic acid; GABA, gamma-aminobutyric acid; IP, prostaglandin I2 receptor; NMDA, N-methyl-D-aspartic acid; TBOB, t-butylbicycloorthobenzoate.
(PDF)

S4 Table Plasma concentration of OX-201 after oral administration in C57BL/6J mice. OX-201 was orally administered to C57BL/6J mice, then blood samples were collected at various time points (0.25, 0.5, 1, 2, 4, 8, and 24 h). Results represent the mean. n = 3. C_max_, maximum concentration; MRT, mean residence time; T_max_, time to reach maximum concentration.
(PDF)

S5 Table Plasma concentration of OX-201 after oral administration in rats. OX-201 was orally administered to rats, then blood samples were collected at various time points (3 mg/kg: 0.5, 1, 2, 3, 4, and 6 h; 10 and 30 mg/kg: 0.25, 0.5, 1, 2, 4, 8, and 24 h). Results represent the mean. n = 3 (3 and 10 mg/kg), n = 4 (30 mg/kg). C_max_, maximum concentration; MRT, mean residence time; T_max_, time to reach maximum concentration.
(PDF)

S6 Table Plasma concentration of OX-201 after intravenous administration in rats. OX-201 was intravenously administered to rats, then blood samples were collected at various time points (0.083, 0.17, 0.25, 0.5, and 1 h). Results represent the mean. n = 5. C_max_, maximum concentration; MRT, mean residence time; T_max_, time to reach maximum concentration.
(PDF)
