# Supplementary figures and images for: Orexin receptor 2 agonist activates diaphragm and genioglossus muscle through stimulating inspiratory neurons in the pre-Bötzinger complex, and phrenic and hypoglossal motoneurons in rodents
Source: PLoS One. 2024 Jun 25;19(6):e0306099. doi: 10.1371/journal.pone.0306099 (PMC11198781; doi:10.1371/journal.pone.0306099)

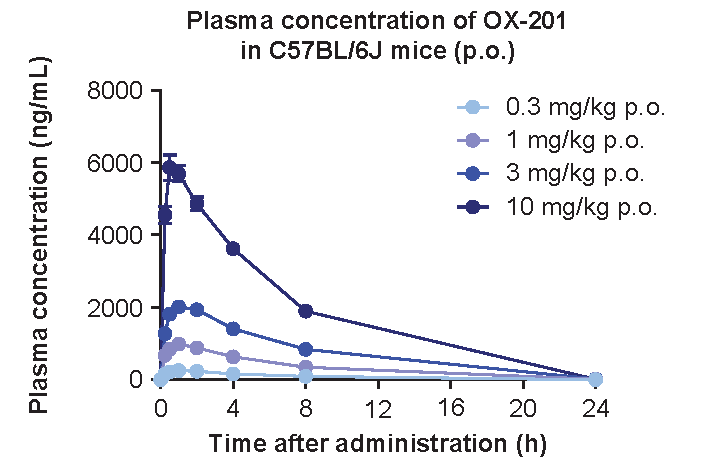

Supplement: S1 Fig — Time-dependent changes in plasma concentrations of OX-201 (p.o.) in C57BL/6J mice. n = 3, mean ± standard error of the mean. (TIF) [file pone.0306099.s001.tif]

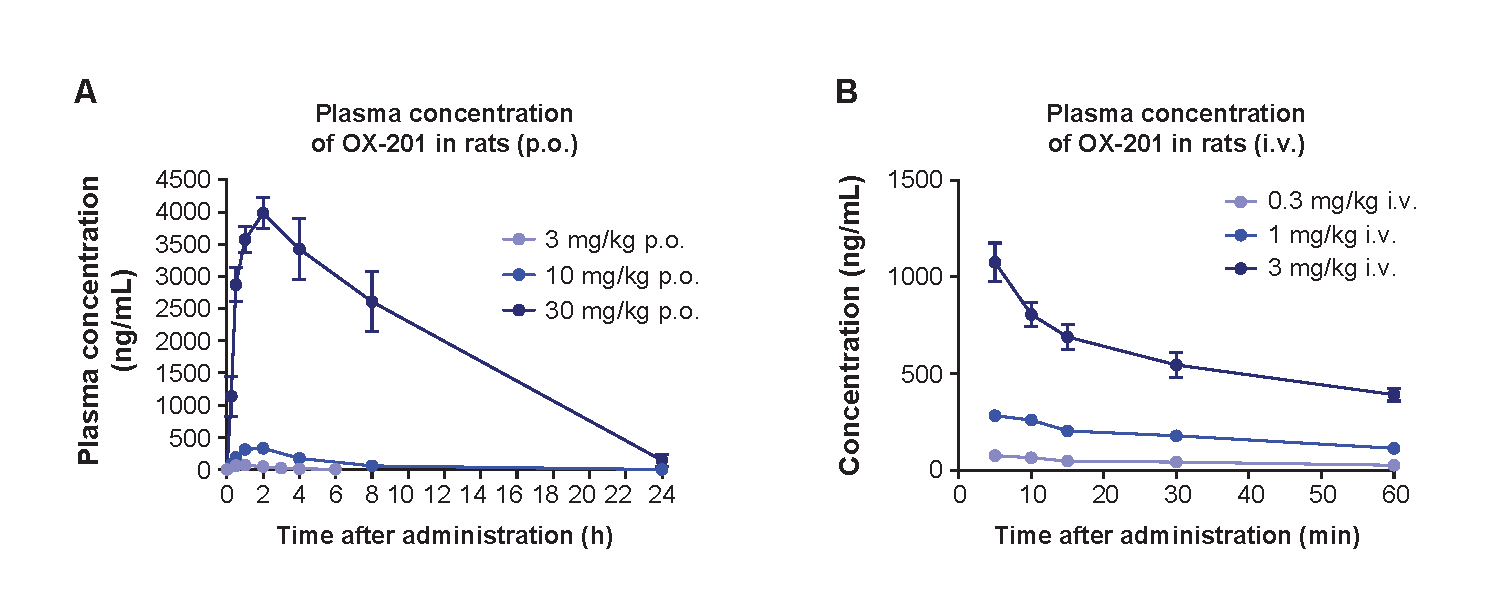

Supplement: S2 Fig — (a) Time-dependent changes in plasma concentrations of OX-201 after oral (p.o.) administration in rats. n = 3–4, mean ± standard error of the mean (SEM). (b) Time-dependent changes in plasma concentrations of OX-201 after intravenous (i.v.) administration in rats. n = 5, mean ± SEM. (TIF) [file pone.0306099.s002.tif]
