# Supplementary material for: Orexin receptor 2 agonist activates diaphragm and genioglossus muscle through stimulating inspiratory neurons in the pre-Bötzinger complex, and phrenic and hypoglossal motoneurons in rodents
Source: PLoS One. 2024 Jun 25;19(6):e0306099. doi: 10.1371/journal.pone.0306099 (PMC11198781; doi:10.1371/journal.pone.0306099)
Supplement: S1 Table — Neuronal activity or burst activity recorded from the cervical ventral root under basal conditions was recorded for 2–20 min, followed by recording in the presence of 0.1% DMSO. DMSO was perfused for 30 min (inspiratory neurons in the pre-Bötzinger complex and hypoglossal motoneurons) or 30–40 min (cervical ventral root). Mean values in the frequency during the last 1–2 min (inspiratory neurons in the pre-Bötzinger complex and hypoglossal motoneurons) or burst frequency during the last 2 min (cervical ventral root) of basal conditions and those during the last 1–2 min (inspiratory neurons in the pre-Bötzinger complex and hypoglossal motoneurons) or 2 min (cervical ventral root) of 0.1% DMSO perfusion were compared. Numbers of recorded neurons or tissues (n) are 8 (inspiratory neurons in the pre-Bötzinger complex), 5 (hypoglossal motoneurons), and 15 (cervical ventral root). Data are presented as mean ± standard error of the mean. DMSO, dimethyl sulfoxide. (PDF) [file pone.0306099.s003.pdf]

	**Inspiratory neurons in the pre-Bötzinger complex**	**Hypoglossal motoneurons**	**Cervical (C3–C5) ventral root**
**Frequency of inspiratory synaptic currents or burst frequency**			
**Basal condition (s^{-1})**	0.14 ± 0.029	0.12 ± 0.026	0.13 ± 0.016
**After 0.1% DMSO perfusion (s^{-1})**	0.14 ± 0.026	0.12 ± 0.029	0.13 ± 0.017
**After 0.1% DMSO perfusion (percent change from basal values)**	-1.21 ± 2.84	2.42 ± 3.96	5.13 ± 2.27
