# Supplementary material for: Orexin receptor 2 agonist activates diaphragm and genioglossus muscle through stimulating inspiratory neurons in the pre-Bötzinger complex, and phrenic and hypoglossal motoneurons in rodents
Source: PLoS One. 2024 Jun 25;19(6):e0306099. doi: 10.1371/journal.pone.0306099 (PMC11198781; doi:10.1371/journal.pone.0306099)
Supplement: S2 Table — ATP, adenosine triphosphate; EGF, epidermal growth factor; HMG-CoA, 3-hydroxy-3-methyl-glutaryl coenzyme A; ROCK1, rho-associated, coiled-coil-containing protein kinase 1. (PDF) [file pone.0306099.s004.pdf]

Enzyme	Percent inhibition
**5-Lipoxygenase**	-2
**ATPase, Ca2+, skeletal muscle**	18
**ATPase, Na+/K+, heart**	13
**Carbonic anhydrase II**	14
**Catechol-O-methyl transferase (COMT)**	-1
**Cholinesterase, acetyl**	5
**Cyclooxygenase (COX)-1**	15
**Cyclooxygenase (COX)-2**	-1
**HMG-CoA reductase**	9
**Monoamine oxidase (MAO)-A**	4
**Monoamine oxidase (MAO)-B**	-5
**Nitric oxide synthase, neuronal (nNOS)**	18
**Nitric oxide synthase, inducible (iNOS)**	-1
**Peptidase, factor Xa**	-3
**Peptidase, matrix metalloproteinase-1 (MMP-1)**	-2
**Peptidase, matrix metalloproteinase-7 (MMP-7)**	-3
**Peptidase, matrix metalloproteinase-13 (MMP-13)**	-1
**Peptidase, metalloproteinase, neutral endopeptidase**	2

**Phosphodiesterase (PDE)10A2**	-1
**Phosphodiesterase (PDE)3**	6
**Phosphodiesterase (PDE)4D2**	5
**Phosphodiesterase (PDE)5**	-14
**Phosphodiesterase (PDE)6**	-6
**Protein serine/threonine kinase, PRKACA (PKA)**	-8
**Protein serine/threonine kinase, PRKCA (PKCα)**	-10
**Protein serine/threonine kinase, ROCK1**	14
**Protein tyrosine kinase, EGF receptor**	18
**Steroid 5α-reductase**	5
**Xanthine oxidase**	2
