# Supplementary material for: Orexin receptor 2 agonist activates diaphragm and genioglossus muscle through stimulating inspiratory neurons in the pre-Bötzinger complex, and phrenic and hypoglossal motoneurons in rodents
Source: PLoS One. 2024 Jun 25;19(6):e0306099. doi: 10.1371/journal.pone.0306099 (PMC11198781; doi:10.1371/journal.pone.0306099)
Supplement: S3 Table — AMPA, α-amino-3-hydroxy-5-methyl-4-isoxazolepropionic acid; GABA, gamma-aminobutyric acid; IP, prostaglandin I2 receptor; NMDA, N-methyl-D-aspartic acid; TBOB, t-butylbicycloorthobenzoate. (PDF) [file pone.0306099.s005.pdf]

Receptors or ion channels	Percent inhibition
**Adenosine A1**	-2
**Adenosine A2A**	6
**Adenosine A2B**	-14
**Adrenergic α1, non-selective**	-9
**Adrenergic α2, non-selective**	4
**Adrenergic β1**	-13
**Adrenergic β2**	7
**Adrenergic β3**	12
**Androgen (testosterone)**	-13
**Angiotensin AT1**	4
**Angiotensin AT2**	11
**Bradykinin B1**	3
**Bradykinin B2**	12
**Calcium channel L-type, benzothiazepine**	29
**Calcium channel L-type, dihydropyridine**	27
**Calcium channel L-type, phenylalkylamine**	20
**Calcium channel N-type**	-9
**Cannabinoid CB1**	51

**Cannabinoid CB2**	14
**Cholecystokinin CCK1 (CCKA)**	-3
**Cholecystokinin CCK2 (CCKB)**	8
**Dopamine D1**	14
**Dopamine D2L**	-6
**Dopamine D2S**	6
**Dopamine D3**	2
**Dopamine D4.4**	-3
**Endothelin ETA**	27
**Estrogen receptor (non-selective)**	-13
**GABAA, chloride channel, TBOB**	-7
**GABAA, flunitrazepam, central**	-9
**GABAA, muscimol, central**	-14
**Glucocorticoid**	9
**Glutamate, AMPA**	22
**Glutamate, kainate**	2
**Glutamate, NMDA, agonism**	5
**Glutamate, NMDA, glycine**	-17
**Glutamate, NMDA, phencyclidine**	-4

**Glycine, strychnine-sensitive**	-9
**Growth hormone secretagogue (GHS, Ghrelin)**	10
**Histamine H1**	5
**Histamine H2**	-20
**Imidazoline I2, central**	-13
**Insulin**	-12
**IP (PGI2)**	-3
**Melatonin MT1**	21
**Muscarinic M1**	-6
**Muscarinic M2**	5
**Muscarinic M3**	9
**Nicotinic acetylcholine α3β4**	-4
**Opiate δ1 (OP1, DOP)**	7
**Opiate κ (OP2, KOP)**	-6
**Opiate μ (OP3, MOP)**	-2
**Potassium channel [KATP]**	-2
**Potassium channel [SKCA]**	12
**Progesterone PR-B**	53
**Serotonin (5-hydroxytryptamine) 5-HT1A**	10

**Serotonin (5-hydroxytryptamine) 5-HT2A**	6
**Serotonin (5-hydroxytryptamine) 5-HT2B**	25
**Serotonin (5-hydroxytryptamine) 5-HT2C**	23
**Serotonin (5-hydroxytryptamine) 5-HT3**	-6
**Serotonin (5-hydroxytryptamine) 5-HT4**	23
**Sigma, non-selective**	26
**Sodium channel, Site 2**	-3
**Tachykinin NK1**	9
**Tachykinin NK2**	-13
**Tachykinin NK3**	-1
**Transporter, dopamine (DAT)**	6
**Transporter, GABA**	3
**Transporter, norepinephrine (NET)**	15
**Transporter, serotonin (5-hydroxytryptamine) (SERT)**	-11
**Transporter, vesicular monoamine (non-selective)**	3
**Vasopressin V1A**	-22
**Vasopressin V2**	-1
