# Supplementary material for: Orexin receptor 2 agonist activates diaphragm and genioglossus muscle through stimulating inspiratory neurons in the pre-Bötzinger complex, and phrenic and hypoglossal motoneurons in rodents
Source: PLoS One. 2024 Jun 25;19(6):e0306099. doi: 10.1371/journal.pone.0306099 (PMC11198781; doi:10.1371/journal.pone.0306099)
Supplement: S4 Table — OX-201 was orally administered to C57BL/6J mice, then blood samples were collected at various time points (0.25, 0.5, 1, 2, 4, 8, and 24 h). Results represent the mean. n = 3. Cmax, maximum concentration; MRT, mean residence time; Tmax, time to reach maximum concentration. (PDF) [file pone.0306099.s006.pdf]

	Oral OX-201			
	**0.3 mg/kg**	**1 mg/kg**	**3 mg/kg**	**10 mg/kg**
**C_{max} (ng/mL)**	265.1	991.3	2081.5	6101.9
**T_{max} (h)**	0.83	1.00	1.33	0.83
**MRT (h)**	5.06	5.17	5.31	4.86
