# Supplementary material for: Orexin receptor 2 agonist activates diaphragm and genioglossus muscle through stimulating inspiratory neurons in the pre-Bötzinger complex, and phrenic and hypoglossal motoneurons in rodents
Source: PLoS One. 2024 Jun 25;19(6):e0306099. doi: 10.1371/journal.pone.0306099 (PMC11198781; doi:10.1371/journal.pone.0306099)
Supplement: S5 Table — OX-201 was orally administered to rats, then blood samples were collected at various time points (3 mg/kg: 0.5, 1, 2, 3, 4, and 6 h; 10 and 30 mg/kg: 0.25, 0.5, 1, 2, 4, 8, and 24 h). Results represent the mean. n = 3 (3 and 10 mg/kg), n = 4 (30 mg/kg). Cmax, maximum concentration; MRT, mean residence time; Tmax, time to reach maximum concentration. (PDF) [file pone.0306099.s007.pdf]

	Oral OX-201		
	**3 mg/kg**	**10 mg/kg**	**30 mg/kg**
**C_{max} (ng/mL)**	71.0	369.3	3995.0
**T_{max} (h)**	1.33	1.67	1.75
**MRT (h)**	1.98	4.00	6.00
