# Supplementary material for: Orexin receptor 2 agonist activates diaphragm and genioglossus muscle through stimulating inspiratory neurons in the pre-Bötzinger complex, and phrenic and hypoglossal motoneurons in rodents
Source: PLoS One. 2024 Jun 25;19(6):e0306099. doi: 10.1371/journal.pone.0306099 (PMC11198781; doi:10.1371/journal.pone.0306099)
Supplement: S6 Table — OX-201 was intravenously administered to rats, then blood samples were collected at various time points (0.083, 0.17, 0.25, 0.5, and 1 h). Results represent the mean. n = 5. Cmax, maximum concentration; MRT, mean residence time; Tmax, time to reach maximum concentration. (PDF) [file pone.0306099.s008.pdf]

	Intravenous OX-201		
	**0.3 mg/kg**	**1 mg/kg**	**3 mg/kg**
**C_{max} (ng/mL)**	76.1	282.1	1075.5
**T_{max} (h)**	0.083	0.083	0.083
**MRT (h)**	0.42	0.43	0.43
